# Inhibition of HDAC2 sensitises antitumour therapy by promoting NLRP3/GSDMD‐mediated pyroptosis in colorectal cancer

**DOI:** 10.1002/ctm2.1692

**Published:** 2024-05-28

**Authors:** Xin Guan, Ruiqi Liu, Bojun Wang, Ruxin Xiong, Luying Cui, Yuanyu Liao, Yuli Ruan, Lin Fang, Xiaolin Lu, Xuefan Yu, Dan Su, Yue Ma, Tianjiao Dang, Zhuo Chen, Yuanfei Yao, Chao Liu, Yanqiao Zhang

**Affiliations:** ^1^ Department of Gastrointestinal Medical Oncology Harbin Medical University Cancer Hospital Harbin China; ^2^ Key Laboratory of Tumor Immunology in Heilongjiang Harbin China; ^3^ Clinical Research Center for Colorectal Cancer in Heilongjiang Harbin China; ^4^ Department of Radiation Oncology Sun Yat‐Sen University Cancer Center Guangzhou China; ^5^ Phase I Clinical Research Center The Affiliated Hospital of Qingdao University Qingdao China; ^6^ Department of Orthopedic Surgery Harbin Medical University Cancer Hospital Harbin China

**Keywords:** colorectal cancer, H3K27ac, HDAC2, NLRP3, pyroptosis

## Abstract

**Background:**

Although numerous studies have indicated that activated pyroptosis can enhance the efficacy of antitumour therapy in several tumours, the precise mechanism of pyroptosis in colorectal cancer (CRC) remains unclear.

**Methods:**

Pyroptosis in CRC cells treated with antitumour agents was assessed using various techniques, including Western blotting, lactate dehydrogenase release assay and microscopy analysis. To uncover the epigenetic mechanisms that regulate NLRP3, chromatin changes and NLRP3 promoter histone modifications were assessed using Assay for Transposase‐Accessible Chromatin using sequencing and RNA sequencing. Chromatin immunoprecipitation‒quantitative polymerase chain reaction was used to investigate the NLRP3 transcriptional regulatory mechanism. Additionally, xenograft and patient‐derived xenograft models were constructed to validate the effects of the drug combinations.

**Results:**

As the core molecule of the inflammasome, NLRP3 expression was silenced in CRC, thereby limiting gasdermin D (GSDMD)‐mediated pyroptosis. Supplementation with NLRP3 can rescue pyroptosis induced by antitumour therapy. Overexpression of HDAC2 in CRC silences NLRP3 via epigenetic regulation. Mechanistically, HDAC2 suppressed chromatin accessibility by eliminating H3K27 acetylation. HDAC2 knockout promotes H3K27ac‐mediated recruitment of the BRD4‐p‐P65 complex to enhance NLRP3 transcription. Inhibiting HDAC2 by Santacruzamate A in combination with classic antitumour agents (5‐fluorouracil or regorafenib) in CRC xenograft‐bearing animals markedly activated pyroptosis and achieved a significant therapeutic effect. Clinically, HDAC2 is inversely correlated with H3K27ac/p‐P65/NLRP3 and is a prognostic factor for CRC patients.

**Conclusion:**

Collectively, our data revealed a crucial role for HDAC2 in inhibiting NLRP3/GSDMD‐mediated pyroptosis in CRC cells and highlighted HDAC2 as a potential therapeutic target for antitumour therapy.

**Highlights:**

- Silencing of NLRP3 limits the GSDMD‐dependent pyroptosis in colorectal cancer.
- HDAC2‐mediated histone deacetylation leads to epigenetic silencing of NLRP3.
- HDAC2 suppresses the NLRP3 transcription by inhibiting the formation of H3K27ac/BRD4/p‐P65 complex.
- Targeting HDAC2 activates pyroptosis and enhances therapeutic effect.

## Graphical abstract

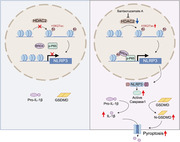

## 1 INTRODUCTION

Colorectal cancer (CRC) contributes to around 10% of both the occurrence and death rates of cancer worldwide.^1^ Roughly half of individuals diagnosed with CRC will eventually progress to metastatic colorectal cancer (mCRC), with chemotherapy and targeted therapy being the primary treatment modalities employed.^2^ Fluorouracil, as the basic agent for chemotherapy, combined with oxaliplatin or irinotecan, can prolong the survival time of patients with mCRC.^3^ Regorafenib, a multitargeted kinase inhibitor, is approved to treat patients with mCRC who exhibit resistance to standard chemotherapy.^4^ Nonetheless, the efficacy rate of combined chemotherapy with 5‐fluorouracil (5‐FU) is below 40%, regorafenib's efficacy rate stands at merely 1.5% and the 5‐year survival rate for patients with mCRC remains below 20%.^5^, ^6^, ^7^ There is an urgent need to increase the efficacy of antitumour therapies for CRC.

Increasing evidence indicates that induction of pyroptosis can significantly enhance therapeutic efficacy against tumours.^8^, ^9^ Pyroptosis is predominantly carried out via the pore‐forming function of gasdermin family proteins, with notable attention given to gasdermin D (GSDMD) and gasdermin E (GSDME), both of which have been extensively studied. Activating GSDMD‐ or GSDME‐driven pyroptosis may become a promising therapeutic approach in various cancers, offering the potential to bypass some of the drug resistance mechanisms often observed in tumour cells.^10^, ^11^ However, GSDME is downregulated or even silenced in CRC.^12^ Therefore, a treatment strategy for activating GSDMD‐mediated pyroptosis is worth exploring.

The classic pyroptosis pathway is mediated by the protein GSDMD.^13^ Upon encountering various stimuli, such as anticancer medications, activation of the NLRP3 inflammasome occurs, resulting in the cleavage of pro‐caspase‐1 into its active form, caspase‐1.^14^ Caspase‐1 activation leads to the cleavage of GSDMD, releasing its N‐terminal fragment, which then oligomerises and creates pores in the plasma membrane. The formation of these pores induces osmotic swelling, cell lysis and discharge of inflammatory cytokines such as interleukin (IL)‐1β and IL‐18. Crucially, several anticancer agents can prompt pyroptosis in tumour cells by stimulating the NLRP3 inflammasome and the subsequent GSDMD pathway.^15^ Pyroptotic cell demise not only directly eliminates cancer cells but also prompts the liberation of inflammatory mediators, which can enhance antitumour immunity and surmount treatment resistance.^16^ A variety of tumours show dysregulated expression of the NLRP3 inflammasome,^17^, ^18^ its expression and regulatory mechanism in CRC remain unclear. CRC exhibits pronounced histone acetylation disturbances, resulting in dysregulation of gene expression.^19^ HDAC2 is frequently elevated in colorectal tumours and associated with tumour progression.^20^ Here, we hypothesised that overexpression of HDAC2 suppresses the level of NLRP3, which limits GSDMD‐mediated pyroptosis in CRC.

In this study, we found that NLRP3 was silent and limited GSDMD‐mediated pyroptosis in CRC. NLRP3 supplementation can reactivate pyroptosis in CRC cells treated with drugs both in vivo and in vitro. Abnormally high HDAC2 expression leads to NLRP3 transcriptional silencing by inhibiting chromatin accessibility. Mechanistically, overexpression of HDAC2 inhibited the formation of the H3k27ac‒BRD4‒p‐P65 complex, which reduced the transcriptional activity of NLRP3. Through in vitro and in vivo experiments, we verified that inhibiting HDAC2 restored NLRP3 expression and facilitated drug‐induced pyroptosis. Our discoveries offer fresh insights into the mechanism by which HDAC2 inhibition triggers pyroptosis in CRC cells, potentially offering valuable clues for CRC therapeutic strategies.

## 2 METHODS

### 2.1 Reagents, antibodies and plasmids

The antibodies utilised in this investigation are detailed in Table S1. The drugs involved in this study include Santacruzamate A (SCA) (S7595, Selleck), regorafenib (S1178, Selleck), 5‐FU (S1209, Selleck), suberoylanilide hydroxamic acid (SAHA; S1047, Selleck), valproic acid (VPA; S3944, Selleck), 5‐Aza (S1782, Selleck), Tazverik (HY‐13803, MedChemExpress), UNC0642 (HY‐13980, MedChemExpress) and KDOAM‐25 (HY‐102047, MedChemExpress). The human GSDMD and NLRP3 overexpression vectors were procured from Vigenebio. Additionally, human NLRP3, HDAC2 and BRD4 siRNAs were obtained from Universal Biology. The pGL‐4.20 vector was kindly provided by the School of Life Science and Technology, Harbin Institute of Technology. The siRNA sequences of NLRP3 were 5′‐AGGAAGAGGAGGAGGAAAATT‐3′ and 5′‐UUUUCCUCCUCCUCUUCCUTT‐3′. The siRNA sequences for HDAC2 were 5′‐GCAUCAGGAUUCUGUUACGTT‐3′ and 5′‐CGUAACAGAAUCCUGAUGCTT‐3′. The siRNA sequences for BRD4 were 5′GGAAAGAGGAAGUGGAAGATT‐3′ and 5′‐UCUUCCACUUCCUCUUUCCTT‐3′.

### 2.2 Patients and samples

Between January 2015 and January 2020, the Harbin Medical University Cancer Hospital provided 200 cases of colorectal tumours and paired adjacent noncancerous tissues. The detailed statistics are provided in Table S2. Following the guidelines set by the medical ethics committee, each participant provided written consent authorising the utilisation of their biological samples and personal data for research objectives. Fresh tumour tissues were collected from two CRC patients to establish patient‐derived xenograft (PDX) models. Fresh tumour tissues were obtained from another two CRC patients to establish patient‐derived organoid (PDO) models.

### 2.3 Lentivirus transduction and CRISPR‒Cas9 gene editing

We used PLent‐Puro‐CMV‐NLRP3 lentiviral vectors (Vigene Biosciences) to clone human NLRP3 sequences. These vectors were subsequently used to package the viral particles. Cells were infected with this virus. Six hours post‐infection, the transduction medium was replaced with the culture medium. The mock‐infected and NLRP3‐overexpressing cells were screened using puromycin (Sigma).

For gene silencing, HDAC2 in CRC cells was targeted using the CRISPR/Cas9 system. According to the instructions, we cloned the HDAC2 guide RNA into the lenti‐CRISPR v2 vector (#52961, Addgene). The constructed plasmid, along with the enhanced green fluorescent protein (eGFP) plasmid, was co‐transfected into SW620 and LS174T cells. GFP‐positive cells were isolated using fluorescence‐activated cell sorting 24 h later, and single‐cell clones were acquired through limited dilution. Individual clones were identified for NLRP3 expression using Western blotting after 14−20 days.

### 2.4 Microscopy imaging

To explore the cellular structure during pyroptosis, LS174T or SW620 cells were plated onto six‐well dishes. Still images of the cells were taken using an OLYMPUS CKX53 microscope designed for cell culture observation. Regorafenib's ability to induce pore formation was assessed through the application of transmission electron microscopy. Cells were subjected to either a 24‐h treatment regimen with 10 µM regorafenib or left untreated as a control group. Following the treatment, the cells were collected, washed with .1 M phosphate‐buffered saline (PBS), and their cell pellets were then fixed in 2.5% glutaraldehyde. Afterward, post‐fixation was conducted using 1% osmium tetroxide, succeeded by dehydration in alcohol, and embedding in epoxy resin for sectioning. Ultrathin sections were stained with uranyl acetate and lead citrate before being subjected to examination using a Hitachi H‐7500 transmission electron microscope.

### 2.5 Immunofluorescence

Initially, cells were washed twice with PBS and then fixed in 4% paraformaldehyde for 10 min at room temperature. Following fixation, cells were permeabilised using 1% Triton‐X100 in PBS for 15 min. Subsequently, the cells were blocked for 45 min at room temperature using a solution containing 3% bovine serum albumin (Sigma) in PBS. After blocking, the cells were incubated with the primary antibody in the blocking solution overnight at 4°C. Subsequently, the cells were washed three times with PBS and incubated with a secondary antibody (Abcam) for 1 h.

Cells were counterstained with DAPI from Selleck, and images were obtained using two microscopy systems: ZEISS LSM 900 with an Airyscan 2 confocal microscope and analysed with ZEN Microscopy Software. The OLYMPUS BX53 upright fluorescence microscope was analysed using the CellSens Standard software.

### 2.6 Immunoprecipitation

SW620 and LS174T cells underwent transfection for a duration of 48 h. Subsequently, they were lysed in NP‐40 lysis buffer (P0013F, Beyotime Biotechnology) supplemented with protease inhibitors (Roche Applied Science) for 30 min at 4°C while placed on a low‐speed rotating shaker. Following this, the lysate was centrifuged at 12 000 revolutions per minute (rpm) and maintained at 4°C for a duration of 10 min. Around 500 µL of primary antibody was introduced, and the mixture was agitated on a rotating shaker at 4°C overnight. Immune complexes were retrieved by incorporating 40 µL of Protein A/G PLUS‐Agarose (sc‐2003, Santa Cruz Biotechnology) and allowing it to incubate for 6 h at 4°C. The immunoprecipitates were gathered through centrifugation at 2500 rpm for 5 min at 4°C. The supernatant was retrieved for subsequent analysis.

### 2.7 Lactate dehydrogenase release assay

The CytoTox96 LDH‐release kit (Promega) was utilised in accordance with the manufacturer's instructions to quantify the release of lactate dehydrogenase (LDH) from cells into the culture supernatants. The release of LDH was verified by measuring absorbance at 490 nm in 96‐well plates. Each experiment was performed in triplicate.

### 2.8 Pyroptosis evaluation by flow cytometry

Pyroptosis was evaluated via flow cytometry. After drug treatment, the cells were harvested and rinsed three times with ice‐cold PBS. Following that, the cells were stained using the Annexin V‐FITC and propidium iodide (PI) Detection Kit (Annexin V‐FITC and PI; Dojindo) as per the manufacturer's instructions. Flow cytometry was utilised to analyse the stained cells within a timeframe of 1 h.

Caspase‐1 activity in CRC cells (LS174T and SW620) was measured by flow cytometry employing a FAM‐FLICA Caspase‐1 Assay Kit (ImmunoChemistry Technologies). Cells were treated with either control (.1% dimethyl sulfoxide (DMSO)), regorafenib (10 µM) or 5‐FU(25 μM). Following treatment, the cells were washed, harvested by centrifugation, and resuspended in medium. The FLICA reagent was then added to stain active Caspase‐1 and incubated for 60 min. Subsequently, PI was introduced to the cell suspension to evaluate cell membrane integrity. After washing, the cells were resuspended once more, and fluorescence intensity was evaluated using flow cytometry to assess the level of active Caspase‐1.

### 2.9 Chromatin immunoprecipitation‒quantitative real‐time polymerase chain reaction

Chromatin purification, immunoprecipitation (IP), qPCR amplification and data analysis were conducted following the protocols outlined in a prior study.^21^ Formaldehyde was used to crosslink chromatin immunoprecipitation (ChIP) DNA‒protein complexes at a concentration of 1%. After removing the impurities, the sonicated extracts were mixed with p‐P65 (RRID:AB_10859369), H3K9ac (RRID:AB_732920), H3K27ac (RRID:AB_2828007) specific, or nonspecific anti‐immunoglobulin G (RRID:AB_2687657) antibodies during incubation. The immunoprecipitated DNA was obtained using phenol/chloroform and subsequently examined by real‐time quantitative polymerase chain reaction (qPCR). Primer details are shown in Table S3.

### 2.10 Tumour xenografts and PDX models

Six‐week‐old female BALB/c‐nude mice (Vital River Laboratories, RRID: MGI:2161072) were subcutaneously injected with 2 × 10^6^ SW620 cells into the right flank. Upon tumour formation, treatment was initiated with either 5‐FU (25 mg/kg, twice a week) or daily regorafenib (30 mg/kg, daily), with the option of adding the HDAC2 inhibitor, SCA (5 mg/kg, daily). Tumour dimensions were consistently monitored every other day using Vernier callipers, and the volume was calculated using the formula: *V* = [length × (width)^2^]/2. Mice were euthanised if the tumour lengths exceeded 1.5 cm in any direction.

For the establishment of the PDX models, please refer to our previous study.^22^ Fresh colon cancer tumour samples were collected promptly after surgical resection and transported in cold complete culture medium. The tissues were then sliced into 2−3 mm pieces and implanted into B‐NSG mice (Biocytogen). Xenograft growth was monitored twice weekly by calliper measurements, with serial passage after tumour volume reached approximately .1 cm^3^. Upon reaching the third passage, mice were randomly allocated to treatment groups once tumours reached approximately .1 cm^3^ in size. The PDX model was maintained in a pathogen‐free environment. Treatment groups received the following: 5‐FU (25 mg/kg, twice a week); regorafenib (30 mg/kg, daily); SCA (5 mg/kg, daily); 5‐FU (25 mg/kg, twice a week) + SCA (5 mg/kg, daily); regorafenib (30 mg/kg, daily) + SCA (5 mg/kg, daily)

### 2.11 Patient‐derived organoid

Immediately after surgical resection, CRC tissues were rinsed with antibiotic‐containing PBS to minimise contamination. Samples were transferred to sterile dishes for processing. Mechanical and Enzymatic Tissue Dissociation Necrotic and adipose tissue was removed. Remaining tissue was minced into uniform pieces using ophthalmic scissors. Tissue fragments were enzymatically digested at 37°C for 30−60 min in a solution of RPMI 1640 with collagenase IV, hyaluronidase and DNase I. The above reagents are all from Sigma‒Aldrich. Digestion was monitored microscopically to ensure dissociation into single cells while preserving viability. The dissociated cell suspension was filtered through 100 and 70 µm strainers, with intermittent centrifugation to collect cells. The final pellet contained the purified tumour cell population. The cell pellet was resuspended in extracellular matrix gel and seeded into 24‐well plates. After solidification, organoid culture medium was added. Organoids were routinely passaged 1:2‒1:6 every 1−2 weeks in freezing medium containing fetal bovine serum (FBS)/DMSO or commercial cryopreservative.

### 2.12 Assay for Transposase‐Accessible Chromatin using sequencing and bioinformatics analysis

Assay for Transposase‐Accessible Chromatin using sequencing (ATAC‐Seq) was conducted on harvested cell samples to characterise genome‐wide open chromatin regions. Briefly, sequencing libraries were prepared using previously described ATAC‐Seq protocols.^23^ To generate paired‐end reads, high‐quality libraries were pooled and sequenced using the Illumina HiSeq system. Initially, the Bowtie2 aligner (RRID:SCR_016368) was employed to align the raw sequencing reads with the human hg38 reference genome. Peak calling was then performed using HOMER (RRID:SCR_010881) to identify the regions of open chromatin in each sample. Merged peaks located within a 500 bp proximity of one another to establish wider regions of accessible chromatin. To characterise the functional roles of these open chromatin regions, peak annotation was performed using the ‘ChIPseeker’ library from R/Bioconductor. Additionally, motif analysis was conducted on peak sequences using HOMER to identify enriched DNA motifs. Downstream analysis involved generating heatmaps and genomic visualisation plots using DeepTools and IGV tools to compare peak profiles across samples. Gene set enrichment analysis (GSEA) (RRID:SCR_005724) was utilised to functionally annotate ranked gene lists displaying accessibility changes. In addition to the GSEA database, gene sets linked to programmed cell death‐related genes were acquired from previous publications and incorporated into the analysis.

### 2.13 RNA sequencing and bioinformatics analysis

Total RNA was extracted and subsequently sequenced utilising an Illumina HiSeq platform to generate paired‐end reads. RNA sequencing (RNA‐Seq) libraries were prepared using the TruSeq kit and sequenced in duplicate. Raw reads were subjected to trimming using Trim Galore (RRID:SCR_011847), followed by alignment to the hg38 reference genome using HISAT2. HTSeq was employed to count the reads mapped to each gene, utilising the RefSeq annotation. Differential expression analysis was performed using DESeq2 (RRID:SCR_000154), which identified differentially expressed genes (DEGs) with a fold change of at least 1.5 and a significance level of *p* < .05. The previously described methods were employed to conduct integration analysis of ATAC‐Seq and RNA‐Seq data, enabling the identification of DEG.^24^ Subsequently, Kyoto Encyclopedia of Genes and Genomes pathway enrichment analysis was carried out on these genes to identify significantly associated pathways. Our study has deposited the raw ATAC‐Seq and RNA‐Seq data in the Genome Sequence Archive database under the accession number HRA005317.^25^

### 2.14 Statistical data

The mean ± standard deviation represents the presentation of quantitative data. GraphPad Prism (RRID:SCR_002798) was employed to assess statistical significance using a two‐tailed unpaired Student's *t*‐test. The analysis of overall survival data utilised Kaplan‒Meier survival curves and log‐rank tests. Statistical significance was defined as a *p*‐value below .05: ^*^ *p* < .05, ^**^ *p* < .01 and ^***^ *p* < .001. A minimum of three independent biological replicates were used for each experiment. *R* values were determined by performing Spearman's correlation analysis.

## 3 RESULTS

### 3.1 Loss of NLRP3 expression in CRC limits GSDMD‐mediated pyroptosis during antitumour therapy

**FIGURE 1:**
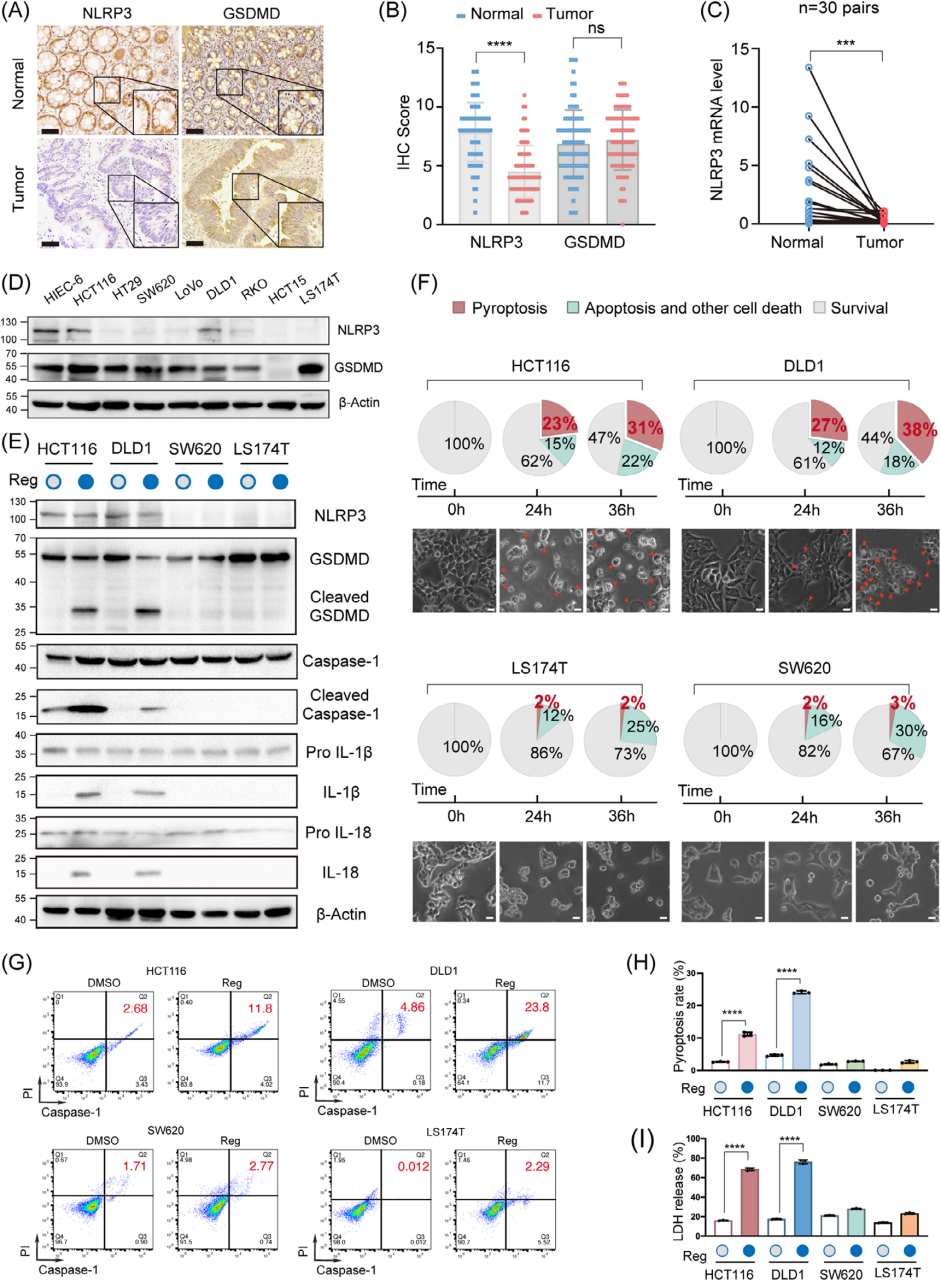
Loss of NLRP3 expression in colorectal cancer (CRC) limits gasdermin D (GSDMD)‐mediated pyroptosis during antitumour therapy. (A and B) Representative immunohistochemistry (IHC) staining images showing NLRP3 and GSDMD expression in CRC tissue (tumour) and corresponding adjacent normal colorectal tissue (normal) from 82 patients. Scale bar: 50 µm. (C) RT‐qPCR analysis demonstrates reduced NLRP3 expression in 30 CRC tissue samples compared to matched adjacent normal tissue samples collected at our institution. (D) Western blot analysis demonstrated the expression levels of the NLRP3 and GSDMD protein in both HIEC‐6 and cancerous cell lines. (E) CRC cells were evaluated for pyroptosis 24 h post‐treatment with regorafenib, as assessed by Western blot. (F) Cell death by regorafenib in CRC cancer cells was morphologically assessed via microscopy. Red arrowheads indicate large bubbles and pores emerging from the plasma membrane. Scale bar: 50 µm. (G and H) Flow cytometry analysis for activated Caspase‐1/propidium iodide (PI). (I) CRC cells were evaluated for pyroptosis 24 h post‐treatment with regorafenib, as assessed by lactate dehydrogenase (LDH) release. Statistical significance is indicated (^*^ *p* < .05, ^**^ *p* < .01, ^***^ *p* < .001, ^****^ *p* < .0001).

Due to the expression of GSDME is deficient across numerous solid tumour types, including CRC (Figure S1A).^12^ To explore pyroptosis in CRC, this study focused on the GSDMD‐mediated pyroptosis pathway. We evaluated the basal expression levels of the key molecule, GSDMD, and its canonical upstream molecule, NLRP3. Immunohistochemical analysis of 82 paired CRC tissues revealed a significant decrease in NLRP3 expression in CRC tissues compared to the corresponding adjacent normal controls, while GSDMD expression remained unchanged (Figure 1A,B). At the mRNA and protein levels, we also demonstrated that NLRP3 expression was decreased in primary CRC tissues (Figures 1C and S1B). Similarly, we observed that NLRP3 was silenced in most CRC cell lines, whereas GSDMD was widely expressed (Figures 1D and S1C). The above results were also validated by analysing transcriptome data obtained from The Cancer Genome Atlas (TCGA) and the GTEx data portal (Figure S1D,E). These findings suggested that NLRP3 expression is silenced in CRC.

To further examine the impact of NLRP3 silencing on pyroptosis in CRC, we selected four CRC cell lines with differential NLRP3 expression to test drug‐induced pyroptosis: HCT116 and DLD1 with relatively high NLRP3 expression, and SW620 and LS174T with relatively low expression (Figure 1D). Western blot analysis showed that NLRP3 silencing inhibited the activation of pyroptotic pathways: less Caspase1, GSDMD cleavage and Pro IL‐1β/IL‐18 release (SW620 and LS174T vs. HCT116 and DLD1) (Figure 1E). Likewise, we observed that NLRP3 knockdown via siRNAs in HCT116 and DLD1 cells abrogated pyroptosis (Figure S2A). After 36 h of regorafenib or 5‐FU treatment, over 30% of NLRP3‐high expressing tumour cells exhibited marked pyroptotic morphologies, including nuclear pyknosis, cellular swelling and membrane blebbing, whereas only approximately 2% of NLRP3‐low expressing cells displayed similar morphological changes (Figures 1F and S2B). Through the implementation of Caspase‐1/PI dual staining in flow cytometry analysis, we observed that treatment with regorafenib or 5‐FU induced a significantly higher percentage of pyroptosis (Caspase‐1+/PI+) in cells expressing high levels of NLRP3 compared to those with low NLRP3 expression (Figures 1G,H and S2C,D). NLRP3‐high expressing tumour cells showed significantly higher LDH release than NLRP3‐low expressing cells, suggesting that high levels of NLRP3 lead to increased cell membrane damage (Figures 1I and S2E). Cell Counting Kit‐8 (CCK‐8) assays in HCT15 cells (where NLRP3 and GSDMD expression was silenced) also demonstrated that co‐expression of key pyroptotic mediators potentiated the cytotoxicity of the drug (Figure S2F). Taken together, these results demonstrate that silencing NLRP3 impaired pyroptosis in CRC cells.

### 3.2 Supplementation of NLRP3 expression in CRC cells rescues GSDMD‐mediated pyroptosis in vivo and in vitro

**FIGURE 2:**
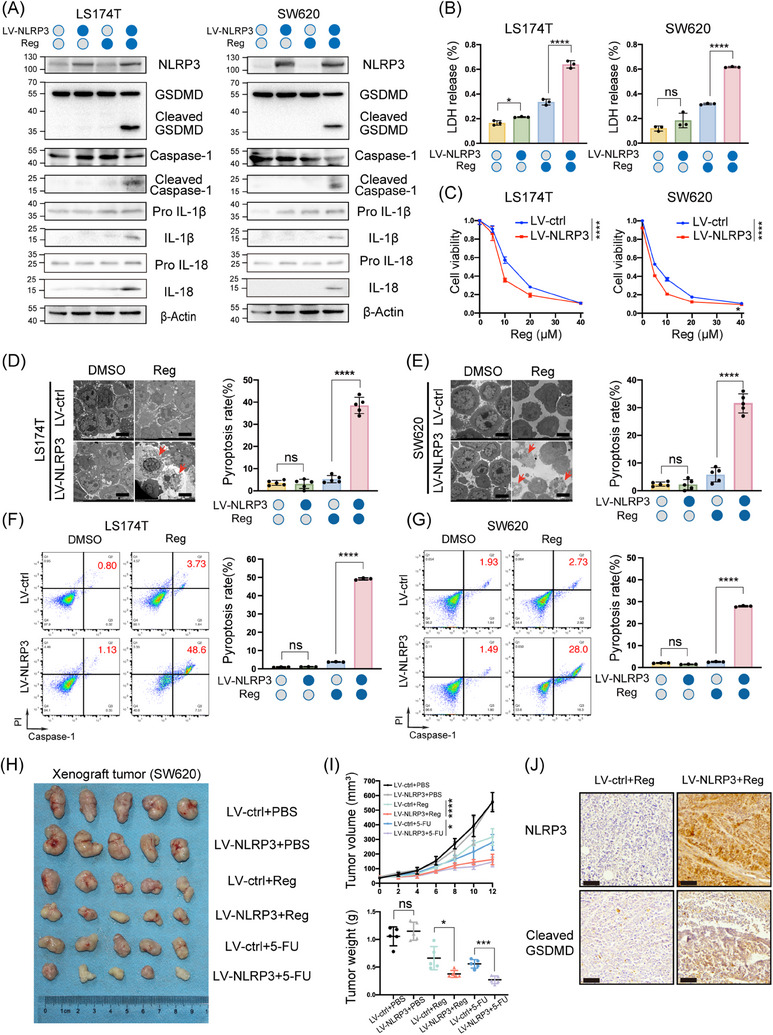
Supplementation of NLRP3 expression in colorectal cancer (CRC) cells rescues gasdermin D (GSDMD)‐mediated pyroptosis in vivo and in vitro. (A and B) Cells stably expressing NLRP3 and control cells were treated with 10 µM regorafenib (Reg) for 24 h. Pyroptosis‐related proteins were assessed by Western blot analysis (A). Lactate dehydrogenase (LDH) release was measured to evaluate pyroptosis levels (B). (C) LS174T and SW620 cells were treated with regorafenib (0, 5, 10, 20 and 40 µM) for 48 h. Cell viability was assessed by CCK‐8 assay. (D and E) Transmission electron microscopy (scale bar: 5 µm) of cells incubated with regorafenib or the corresponding control. Red arrowheads indicate pyroptosis. (F and G) Flow cytometry analysis for activated Caspase‐1/propidium iodide (PI). (H) SW620 xenografts were established in nude mice and treated as shown (*n* = 5/group). (I) Tumour growth curves were generated from volumes recorded every 2 days. Tumour weight was measured on day 12 after drug treatment. (J) The expression of NLRP3 and cleaved GSDMD in xenografts from different treatment groups was analysed by immunohistochemistry (IHC). Scale bar: 60 µm. Statistical significance is indicated (^*^ *p* < .05, ^**^ *p* < .01, ^***^ *p* < .001, ^****^ *p* < .0001).

Given the frequent silencing of NLRP3 in CRC, we investigated the effect of NLRP3 re‐expression on pyroptosis. While NLRP3 overexpression did not affect the cleavage of the pyroptotic markers GSDMD, Pro IL‐1β, Pro IL‐18 and Caspase‐1 compared to wild‐type cells, significant increases were observed following treatment with various chemotherapeutic drugs such as regorafenib or 5‐FU (Figures 2A and S3A). Moreover, NLRP3 overexpression enhanced multiple drug‐induced LDH release and reduced cell viability in CRC cells compared to that in the controls (Figures 2B,C and S3B,C). Microscopically, NLRP3‐overexpressing cells displayed distinct balloon‐like bubbles after regorafenib treatment, which were absent in controls (Figure S3D,E). To further characterise these structures, high‐resolution scanning electron microscopy was used to analyse the damaged NLRP3‐overexpressing cells. Ultrastructural examination revealed more vigorous membrane protrusions (Figure 2D,E). Annexin V is a protein that specifically binds to phosphatidylserine, a phospholipid normally located on the inner leaflet of the plasma membrane in healthy cells. PI is a DNA‐intercalating dye that typically cannot penetrate intact cell membranes but can enter cells with compromised membranes, such as those undergoing cell death. Flow cytometry showed that NLRP3 overexpression led to a gradual increase in the proportion of cells positive for both PI and Annexin V compared to that in the control group (Figure S4A,B). To enhance the specificity of pyroptosis detection by flow cytometry, Caspase‐1/PI flow cytometry assay was assessed, revealing significantly increased percentage of pyroptosis (Caspase‐1+/PI+) in NLRP3‐overexpressing cells compared to that in controls (Figures 2F,G and S4C,D).

To further elucidate the role of NLRP3 re‐expression in promoting pyroptosis in CRC in vivo, we utilised a subcutaneous xenograft tumour model by implanting NLRP3‐overexpressing SW620 cells. Over a 12‐day period, NLRP3‐overexpressing tumours displayed attenuated tumour growth and reduced final tumour burden in response to regorafenib or 5‐FU treatment, compared to control xenografts (Figure 2H,I). Immunohistochemistry (IHC) and Western blot analyses of NLRP3‐overexpressing xenografts revealed elevated intratumoural levels of pyroptosis‐associated markers (Cleaved caspase‐1 and cleaved GSDMD) relative to control tumours after sustained drug administration (Figures 2J and S4E). To align with clinical practice, we investigated the impact of NLRP3 re‐expression on the efficacy of combined treatment with 5‐FU and oxaliplatin. In the xenograft tumour model, NLRP3‐overexpressing tumours were significantly smaller than wild‐type tumours under combined treatment with 5‐FU and oxaliplatin (Figure S4F,G). Taken together, these in vivo data corroborate our in vitro findings that NLRP3 re‐expression facilitates pyroptosis in CRC cells, highlighting NLRP3 as a key mediator of pyroptotic cell death in this setting.

### 3.3 HDAC2‐mediated histone deacetylation leads to epigenetic silencing of NLRP3 in CRC

**FIGURE 3:**
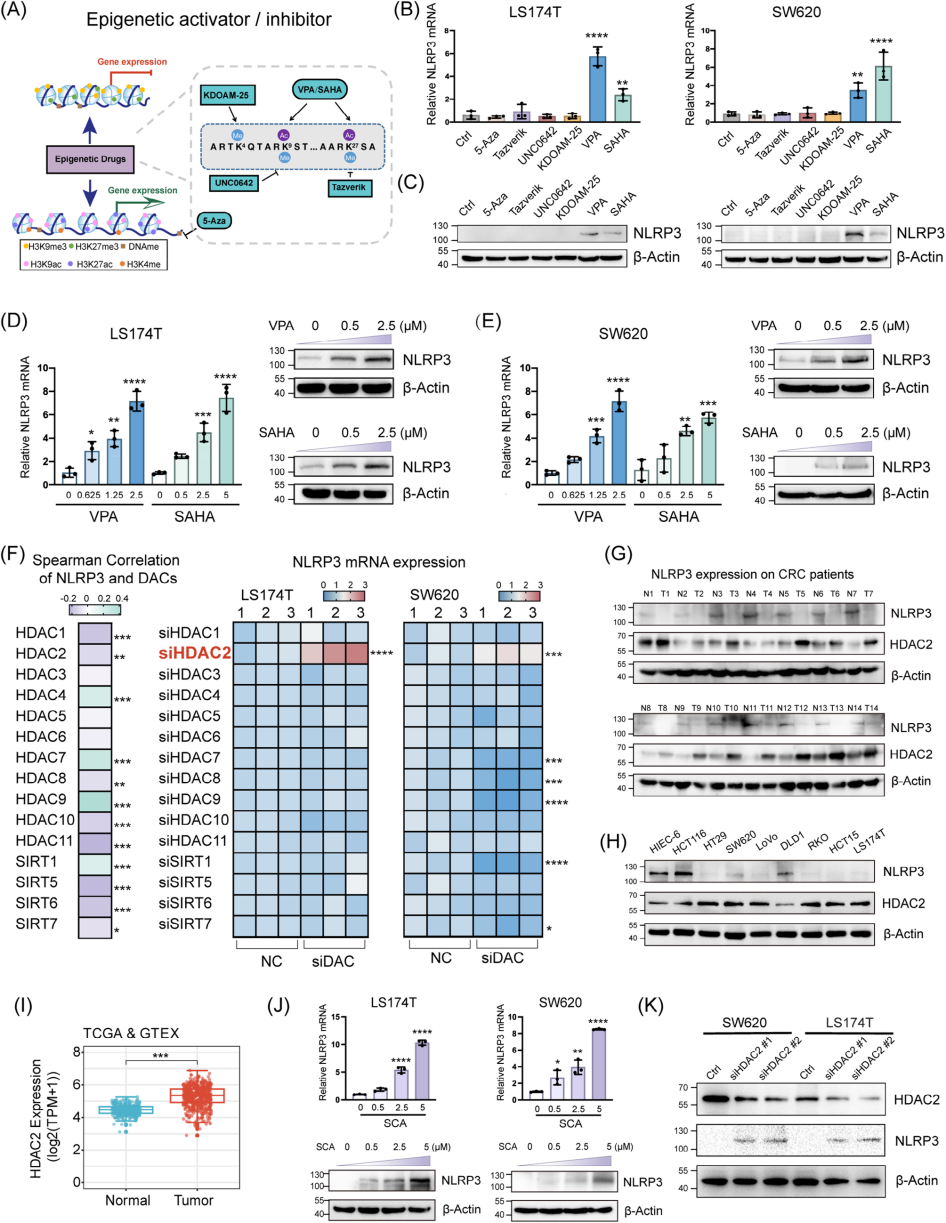
HDAC2‐mediated histone deacetylation leads to epigenetic silencing of NLRP3 in colorectal cancer (CRC). (A) Overview of the general mechanisms and canonical target sites for various epigenetic drugs. (B and C) Multiple epigenetic drugs were applied to LS174T and SW620 cells. RT‐qPCR and Western blotting were used to analyse the changes in the mRNA and protein levels of NLRP3, respectively. (D and E) RT‐qPCR and Western blot analyses were conducted to assess the expression levels of NLRP3 in LS174T and SW620 cells after exposure to escalating doses of histone deacetylase (HDAC) pan‐inhibitors. Two distinct small molecule HDAC pan‐inhibitors were utilised independently. (F) Profiling of HDAC enzymes influencing NLRP3 transcription. Spearman correlation analysis was performed between 15 deacetylases (DACs) and NLRP3 using The Cancer Genome Atlas (TCGA) data. RNA interference was used to individually knockdown 15 DACs, and RT‐qPCR was performed to assess the effects on NLRP3 gene expression. (G) Western blot analysis of NLRP3 and HDAC2 protein levels in 14 matched pairs of CRC tissues and neighbouring normal tissues. (H) Western blot analysis demonstrated the expression levels of the NLRP3 and HDAC2 protein in both HIEC‐6 and CRC cells. (I) A joint analysis of HDAC2 expression in CRC cancer tissues and their corresponding normal tissues was conducted using data from the TCGA and GTEx databases. (J) RT‐qPCR and Western blot analyses were conducted to assess the expression levels of NLRP3 in CRC cells after exposure to escalating doses of Santacruzamate A (SCA). (K) NLRP3 protein expression in LS174T and SW620 cells was detected by Western blot following HDAC2 knockdown. Statistical significance is indicated (^*^ *p* < .05, ^**^ *p* < .01, ^***^ *p* < .001, ^****^ *p* < .0001).

Silencing of NLRP3 impairs pyroptosis in CRC; however, the precise mechanisms underlying NLRP3 silencing remain poorly defined. CRC exhibits substantial epigenetic instability, which manifests as the silencing of tumour suppressor genes due to hypermethylation of CpG islands in the promoter region, changes in histone modifications, and dysregulation of epigenetic modifiers that contribute to tumour initiation and progression. Therefore, we aimed to explore the key drivers of epigenetic NLRP3 silencing in CRC. Active histone marks such as H3K4me, H3K9ac and H3K27ac and repressive marks such as H3K9me3 and H3K27me3 are modulated by various histone‐modifying enzymes, whereas DNA methylation is catalysed by DNA methyltransferases. A schematic illustrates the roles of different epigenetic drugs and their sites of action (Figure 3A). Screening of the above epigenetic drugs identified SAHA and VPA as potent inducers of NLRP3 in CRC cells, significantly elevating transcript and protein expression (Figure 3B,C). SAHA and VPA are both histone deacetylase (HDAC) pan‐inhibitors, targeting class I and II HDACs. The result preliminarily determined that the epigenetic mechanism regulating NLRP3 expression is histone acetylation. Further results demonstrated that SAHA and VPA dose dependently increased NLRP3 levels in four colon cancer cell lines (Figures 3D,E and S5A,B). The failure of DNA methyltransferase inhibitors to re‐establish NLRP3 expression in our epigenetic drug screen, combined with database evidence showing hypomethylation at the NLRP3 promoter region in tumours (Figure S5C), suggests that DNA methylation is not accountable for the transcriptional silencing of NLRP3. Collectively, our findings suggest that the deacetylase inhibitors SAHA and VPA induce NLRP3 re‐expression by inhibiting HDAC activity, underscoring HDAC‐mediated chromatin condensation as a mechanism underlying NLRP3 repression.

Given that VPA and SAHA broadly target HDACs, subsequent experiments focused on identifying the specific HDAC family members that mediate epigenetic modification of NLRP3. Spearman's correlation coefficient analysis unveiled a noteworthy negative correlation between NLRP3 and the majority of HDACs (Figure 3F). Concurrently, siRNA screening of these deacetylases showed that only HDAC2 knockdown increased NLRP3 transcription in CRC cells (Figure 3F). Patient‐derived CRC tissue exhibited heightened HDAC2 protein abundance relative to normal adjacent tissues, as assessed by immunoblotting, which was negatively correlated with NLRP3 levels (Figure 3G). Furthermore, Western blot analysis of CRC cell lines corroborated this intriguing inverse relationship between HDAC2 and NLRP3 expression (Figure 3H). The analysis of TCGA data revealed that HDAC2 expression was upregulated in CRC samples compared to normal samples (Figure 3I). The result of western demonstrate that activation of the Wnt pathway using LiCl induces a gradual increase in HDAC2 protein expression, paralleling the rise in β‐catenin levels (Figure S5D). Following JASPAR prediction analysis, the data revealed the presence of a c‐Myc binding motif within the promoter region of the HDAC2 gene (Figure S5E). CHIP‒qPCR assays further confirm that c‐Myc mediates the transcriptional activation of HDAC2 upon LiCl treatment (Figure S5F). Our findings are consistent with previous studies, confirming that abnormal activation of the Wnt pathway leads to high expression of HDAC2 in CRC. Notably, treatment with SCA, a selective HDAC2 inhibitor, induced a dose‐dependent upregulation of NLRP3 levels in colon cancer cells (Figure 3J). Compared to non‐targeting siNC transfection, siHDAC2 transfection significantly increased the protein levels of NLRP3 (Figure 3K). These results suggest that abnormally high expression of HDAC2 in CRC leads to the epigenetic silencing of NLRP3.

### 3.4 Knocking out HDAC2 activates GSDMD‐mediated pyroptosis by upregulating NLRP3

**FIGURE 4:**
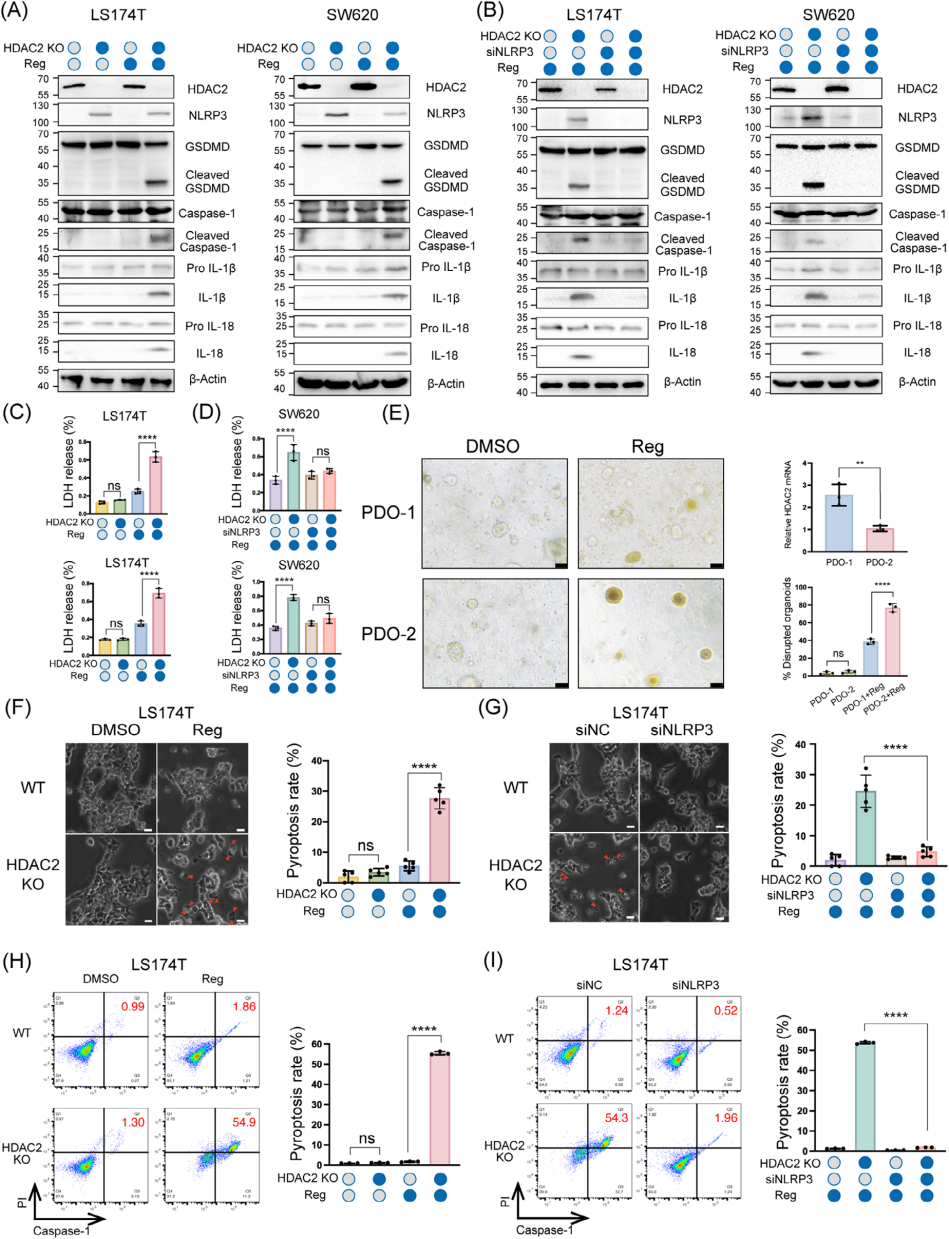
Knocking out HDAC2 activates gasdermin D (GSDMD)‐mediated pyroptosis by upregulating NLRP3. (A‒D) LS174T and SW620 HDAC2 knockout lines were generated with CRISPR/Cas9. Treated with 10 µM regorafenib, and transfected with NLRP3 siRNA in knockout cells for rescue experiments. Pyroptosis pathway proteins were analysed by Western blotting (A and B), and pyroptosis was evaluated by measuring lactate dehydrogenase (LDH) release (C and D). (E) Organoids were treated with regorafenib (10 µM), and the percentage of organoids with changed morphology was quantified using light microscopy. Scale bar: 50 µm. (F and G) Typical bright‐field microscopy images of LS174T cells are shown. Large bubbles protruding from the plasma membrane are highlighted by red arrows. Scale bar: 50 µm. Dead cells were tallied and quantified based on five separate images. (H and I) Flow cytometry analysis for activated Caspase‐1/propidium iodide (PI). Statistical significance is indicated (^*^ *p* < .05, ^**^ *p* < .01, ^***^ *p* < .001, ^****^ *p* < .0001).

Our findings demonstrate that NLRP3 silencing inhibits pyroptosis, while aberrantly elevated HDAC2 epigenetically suppresses NLRP3 in CRC. Therefore, we evaluated whether HDAC2 regulated drug‐induced pyroptosis by modulating NLRP3 expression. SW620 and LS174T CRC cells with stable CRISPR/Cas9‐mediated HDAC2 knockout (KO) were generated and treated with regorafenib or 5‐FU for 24 h. Western blot analysis verified that HDAC2 deletion resulted in a Cleaved GSDMD and IL‐1β (Figures 4A and S6A). However, NLRP3 knockdown in HDAC2‐deficient cells attenuated cleavage of these proteins (Figure 4B). LDH levels were increased in HDAC2 KO cells compared with control cells and could also be reversed by knockdown of NLRP3 (Figure 4C,D). Furthermore, CCK‐8 assays revealed increased drug sensitivity in HDAC2‐deficient cells, which was rescued by simultaneous NLRP3 knockdown (Figure S6B). To comprehensively validate these findings, we performed experiments using PDO cultures with differential HDAC2 expression levels. Upon exposure to the regorafenib, organoids with lower HDAC2 expression (PDO‐2) exhibited more pronounced morphological disruption compared to those with higher HDAC2 expression levels (PDO‐1) (Figure 4E).

Optical microscopy revealed that approximately 30%−40% of HDAC2 KO colon cancer cells displayed pyroptotic morphologies (characteristic ballooning of the cell membrane) after drug treatment, compared to only 3%−6% of wild‐type cells (Figures 4F and S6C). However, siRNA‐mediated knockdown of NLRP3 in HDAC2 KO lines markedly reduced the proportion of pyroptotic cells to less than 10% (Figures 4G and S6D). Annexin V/PI staining corroborated the significantly increased uptake of Annexin V and PI in HDAC2‐depleted versus wild‐type cells (60%−70% vs. 30%−40%), which was also attenuated by NLRP3 knockdown (Figure S7A‒D). To enhance the accuracy of detecting pyroptosis, we implemented Caspase‐1 and PI double staining. The results revealed that the level of regorafenib or 5‐FU‐induced pyroptosis increased upon HDAC2 KO, while the pyroptosis level decreased after NLRP3 knockdown (siNLRP3) (Figures 4H,I and S7E,F). In addition, we also examined Caspase‐3 activity to preliminarily assess the impact of HDAC2 on apoptosis. The results indicated that there was no significant difference in drug‐induced apoptosis levels between HDAC2 KO cells and control cells (Figure S7G). Using an orthotopic model of CRC, we found that HDAC2 KO tumours were more sensitive to treatment with regorafenib or 5‐FU than wild‐type tumours (Figure S7H). In summary, our results identified NLRP3 as an integral functional target of HDAC2 and substantiated the NLRP3 pathway, in which HDAC2 governs pharmacological pyroptosis in colorectal carcinoma by modulating NLRP3 levels.

### 3.5 HDAC2 blocks the transcriptional effect of p‐P65 on NLRP3 by inhibiting chromatin accessibility

**FIGURE 5:**
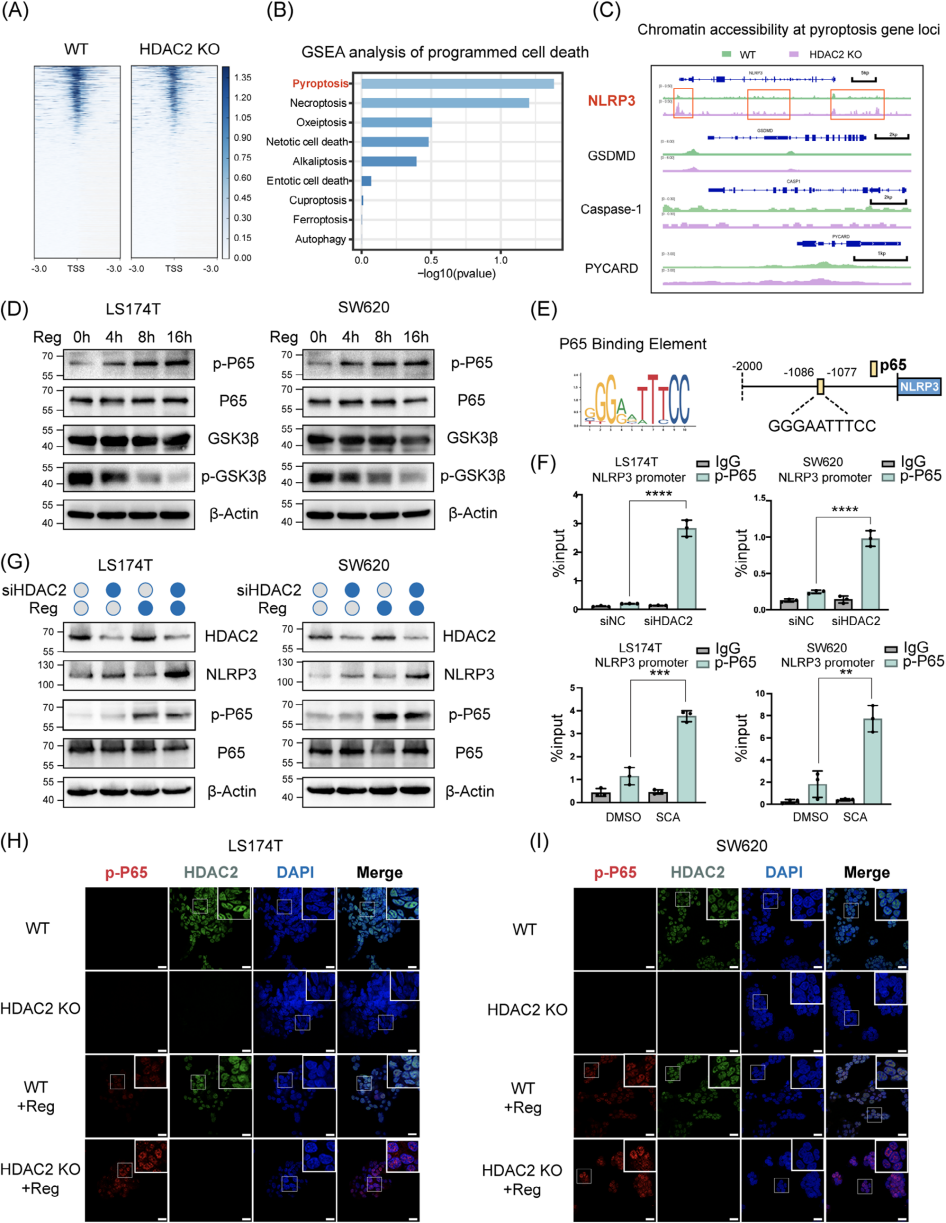
HDAC2 blocks the transcriptional effect of p‐P65 on NLRP3 by inhibiting chromatin accessibility. (A)  Heatmap showing the average ATAC‐Seq signal centred on the transcription start site (TSS) of the genes in HDAC2 knockout (KO) or wild‐type SW620. The regions of enrichment were expanded by 3 kb on either side of their central point. (B) Gene set enrichment analysis (GSEA) software was used to perform functional enrichment analysis on ranked gene sets of accessibility changes related to various forms of programmed cell death. (C) Genome browser visualisation of ATAC‐Seq data for the NLRP3, gasdermin D (GSDMD), Caspase‐1 and PYCARD Loci in SW620 cells following HDAC2 silencing. (D) The levels of total GSK3β, p‐GSK3β (Ser9), total P65 and p‐P65 (Ser536) were analysed by Western blotting in LS174T or SW620 cells treated with 10 µM regorafenib at the indicated time points. (E) Schematic representation illustrating the positions of potential P65 binding sites in the promoter region of the NLRP3 gene. (F) Chromatin immunoprecipitation (ChIP)‒qPCR validated the interaction between p‐P65 and the NLRP3 promoter in LS174T and SW620 cells under conditions with or without HDAC2 knockdown, as well as with or without Santacruzamate A (SCA) treatment. (G) Expression of NLRP3, HDAC2, P65 and p‐P65 was analysed by Western blotting in LS174T and SW620 cells treated with 10 µM regorafenib in the presence or absence of HDAC2 knockdown. (H and I) The subcellular localisation of p‐P65 and HDAC2 was assessed by double immunofluorescence staining using their respective antibodies. Images were acquired using a confocal microscope. Scale bar: 20 µm. Statistical significance is indicated (^*^ *p* < .05, ^**^ *p* < .01, ^***^ *p* < .001, ^****^ *p* < .0001).

The class I HDAC family member HDAC2 contains a catalytic deacetylase domain that mediates histone deacetylation, leading to NLRP3 closed chromatin structure and transcriptional repression. To investigate the mechanism by which HDAC2 mediates the silencing of NLRP3, we studied genome‐wide chromatin accessibility changes regulated by HDAC2 in wild‐type or HDAC2 KO SW620 cells using ATAC‐Seq. As shown in the heatmap, HDAC2 silencing did not significantly alter overall chromatin accessibility in SW620 cells (Figure 5A). To characterise the roles of HDAC2 in regulating cell death, we implemented GSEA on genes ranked by changes in chromatin accessibility following HDAC2 KO. The results showed that pyroptosis pathways were significantly enriched, ranking first (Figure 5B). Browser visualisation of chromatin accessibility changes revealed heightened open chromatin architecture, specifically at the NLRP3 promoter, without similar effects at the regulatory regions of other pyroptotic genes GSDMD, Caspase‐1 and PYCARD (Figure 5C). In parallel, we performed RNA‐Seq on wild‐type and HDAC2 KO SW620 cells. Integration of RNA‐Seq and ATAC‐Seq data demonstrated the activation of the NOD‐like receptor signalling pathway, which includes NLRP3 (Figure S8A). These results demonstrate that HDAC2 KO activates the pyroptosis pathway and increases chromatin accessibility in the NLRP3 promoter region.

Next, we sought to elucidate the transcriptional mechanisms involved in the restoration of NLRP3 expression. In addition to the NOD‐like pathway, integrative analysis also revealed the activation of nuclear factor kappa B (NF‐κB) signalling, a canonical NLRP3 transcriptional pathway with the transcription factor p‐P65 driving NLRP3 expression (Figure S8A). Various agents can engage in the NF‐κB signalling cascade to drive gene expression. Previous studies have shown that regorafenib significantly increases p‐P65 (Ser536) activation by inhibiting GSK3β (Ser9) autophosphorylation, consistent with our data (Figure 5D). Concurrently, we found that 5‐FU similarly stimulated p‐P65 activation (Figure S8B). Additionally, to comprehensively profile the drug‐triggered transcriptional mechanisms, we tested luciferase reporters for known NLRP3 transcription factors, including NF‐κB, STAT1, STAT3, NFATC1 and AP‐1 (Figure S8C). Among these, only the NF‐κB reporter was upregulated by the drug treatment. These findings indicate that drug exposure stimulates the canonical NLRP3 transcription factor P65 in CRC.

Having shown that HDAC2 inhibition increased chromatin accessibility at the NLRP3 promoter, while drug treatment activated the classical NLRP3 transcription factor NF‐κB, we next sought to validate that these mechanisms combinatorially restored robust NLRP3 expression under drug stimulation. After examining the DNA sequence of the NLRP3 promoter, a putative binding site for the transcription factor P65 was identified. This site, positioned −1086 to −1077 bp (GGGAATTTCC) upstream of the NLRP3 transcription start site, was detected (Figure 5E). Subsequent ChIP assays demonstrated heightened binding of the transcription factor p‐P65 to the NLRP3 promoter region following either HDAC2 KO or treatment with the HDAC2‐specific inhibitor SCA (Figure 5F). Furthermore, SCA treatment diminished the binding of HDAC2 to the promoter region of the NLRP3 gene, which did not occur with 5‐FU treatment (Figure S9A). Western blot analysis revealed a significant increase in NLRP3 levels in HDAC2 KO cells treated with regorafenib (Figure 5G). Immunofluorescence also showed that regorafenib could activate P65 phosphorylation and nuclear import, which was enhanced in HDAC2 KO cells (Figures 5H,I and S9B,C). Moreover, we utilised an in vivo lung metastasis model to assess the effects of targeting the NF‐κB pathway, alongside anticancer treatment. Dehydroxymethylepoxyquinomicin (DHMEQ), a p65 inhibitor blocking its nuclear translocation, was employed. Notably, NF‐κB inhibition via DHMEQ abrogated the increased treatment sensitivity observed upon HDAC2 KO, validating that HDAC2 mediates pyroptosis in a P65‐dependent manner (Figure S9D,E). These results demonstrated that HDAC2 KO heightened chromatin accessibility at the NLRP3 promoter to allow more p‐P65 recruitment under drug‐induced conditions, promoting NLRP3 transcriptional activation.

### 3.6 Inhibition of HDAC2 activates NLRP3 transcription by facilitating the formation of the H3K27ac‒BRD4‒p‐P65 complex

**FIGURE 6:**
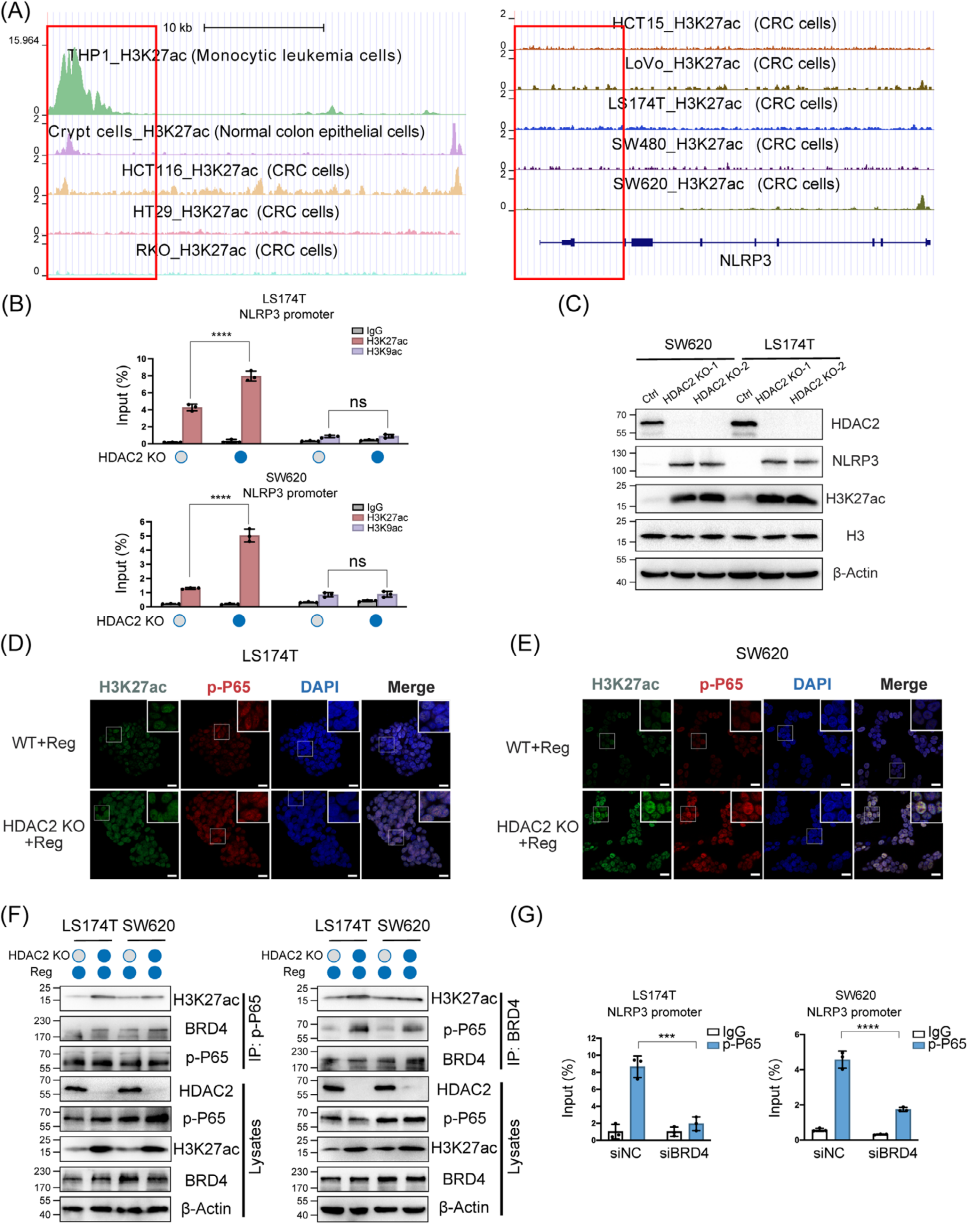
Inhibition of HDAC2 activates NLRP3 transcription by facilitating the formation of the H3K27ac‐BRD4‒p‐P65 complex. (A) Chromatin immunoprecipitation (ChIP)‐seq analysis of H3K27ac binding to the NLRP3 promoter region in various cells as predicted by Cistrome DataBrowser and visualised by UCSC Genome Browser. H3K27ac marks active chromatin. (B) ChIP‒qPCR analysis of the binding between the indicated histone proteins (H3K9ac and H3K27ac) and the NLRP3 promoter in colorectal cancer (CRC) wild‐type or HDAC2 knockout (KO) cells. Total genomic DNA and nonspecific immunoglobulin G were the input and negative antibody controls, respectively. (C) Protein levels of NLRP3 and H3K27ac were detected in LS174T cells and SW620 cells after HDAC2 KO. Two independent HDAC2 KO cell lines were provided each for either LS174T cells or SW620 cells. (D and E) The subcellular localisation of p‐P65 and HDAC2 was examined by double immunofluorescence staining with their corresponding antibodies. Images were obtained using confocal microscopy. Scale bar: 20 µm. (F) Co‐immunoprecipitation (Co‐IP) and immunoblotting showing the effect of HDAC2 knockdown on p‐P65 binding with BRD4/H3K27ac and BRD4 binding with p‐P65/H3K27ac in regorafenib‐treated CRC cells. (G) ChIP‒qPCR analysis of the binding between p‐P65 and the NLRP3 promoter in HDAC2 KO LS174T and SW620 cells under normal or BRD4 knockdown conditions. Statistical significance is indicated (^*^ *p* < .05, ^**^ *p* < .01, ^***^ *p* < .001, ^****^ *p* < .0001).

Having demonstrated that HDAC2 ablation permitted more robust p‐P65 recruitment and subsequent NLRP3 transcriptional activation, we next conducted experiments to further delineate the precise regulatory dynamics allowing enhanced p‐P65 recruitment to the NLRP3 promoter after HDAC2 KO. Specifically, HDAC2 has been shown to deacetylate histone H3 lysine 27 (H3K27ac) and H3 lysine 9 (H3K9ac), both of which are associated with open, active chromatin and transcriptional activation. THP‐1 cells expressing high levels of NLRP3 are a well‐established human monocytic leukemia cell line that has been extensively used as a model system to study pyroptosis. In addition to THP‐1 cells, we also included colonic crypt cells, which are normal intestinal epithelial cells, as a tissue‐specific control for our CRC cell lines. By analysing publicly available ChIP‐seq datasets, we observed that H3K27 acetylation levels within the NLRP3 promoter region are significantly lower in CRC cells compared to both THP‐1 cells and colonic crypt cells (Figure 6A). These ChIP‐seq profiles were concordant with changes in chromatin openness determined by ATAC‐Seq, implying that hypoacetylation of H3K27 leads to an NLRP3 closed chromatin architecture at the NLRP3 locus (Figure S10). To validate this, we performed ChIP for H3K27ac and H3K9ac in HDAC2 KO versus control cells. ChIP revealed increased H3K27ac, but not H3K9ac, at the promoter of the NLRP3 gene upon HDAC2 deletion (Figure 6B). Thus, HDAC2 appears to regulate H3K27 acetylation to modulate NLRP3 gene transcription. Western blot analysis revealed increased H3K27ac levels and correspondingly higher expression of NLRP3 in HDAC2 KO cells than in controls (Figure 6C). This indicates that HDAC2 specifically deacetylates H3K27ac, rather than H3K9ac, to silence NLRP3.

Fluorescence microscopy analysis revealed increased nuclear H3K27 acetylation in HDAC2 KO cells compared to wild‐type controls, as well as enhanced p‐P65 nuclear recruitment in HDAC2 KO cells following regorafenib treatment (Figure 6D,E). Moreover, H3K27ac displayed nuclear colocalisation with p‐P65 in the cells. Prior research has established that acetylated H3K27 acts as an anchor for BRD4, which then bridges the connection between chromatin and P65 to stimulate the transcription of downstream genes. To validate the interactions between H3K27ac, BRD4 and P65, we performed co‐immunoprecipitation experiments in cells and observed that endogenous BRD4 forms an NLRP3 complex with P65 and H3K27ac, which was enhanced upon HDAC2 KO (Figure 6F). Additionally, ChIP with a p‐P65 antibody demonstrated that p‐P65 occupancy at the NLRP3 promoter decreased after BRD4 siRNA treatment inhibition in HDAC2 KO CRC cells (Figure 6G). In summary, our results revealed that HDAC2 loss increased H3K27ac, promoting the formation of H3K27ac‒BRD4‒p‐P65 complexes to recruit more p‐P65 and drive NLRP3 transcriptional activation.

### 3.7 HDAC2 correlates with H3K27ac/p‐P65/NLRP3 predicts prognosis and represents a promising target in CRC

**FIGURE 7:**
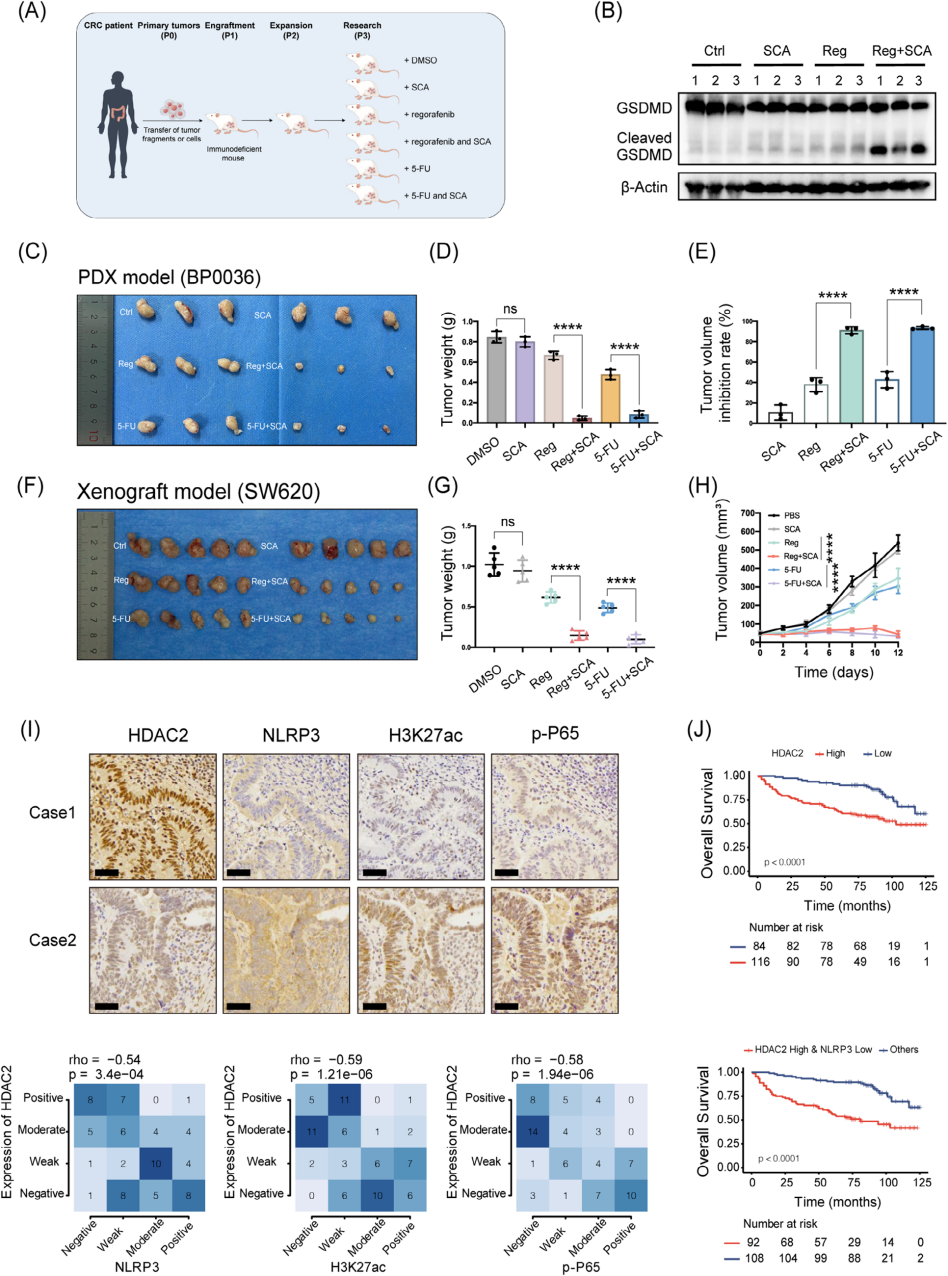
HDAC2 correlates with H3K27ac/p‐P65/NLRP3, predicts prognosis and represents a promising target in colorectal cancer (CRC). (A) A schematic diagram outlines the workflow for establishing patient‐derived xenograft (PDX) models of CRC. To evaluate therapeutic strategies, mice bearing established PDX tumours were then randomly divided into six treatment groups as depicted in the schematic. (B) Expression of gasdermin D (GSDMD) and Cleaved GSDMD in tumour tissues from BP0036 mice under different treatment conditions was analysed by Western blotting. (C and D) In the CRC PDX model BP0036, which exhibits high expression of HDAC2, tumour size (C) and weight (D) are shown after grouping and 12 days of treatment. (E) Tumour growth inhibition rate of each PDX model under different treatment regimens. (F and G) The xenograft tumour size (F) and weight (G) were assessed approximately 12 days after initiating treatment. (H) Tumour volumes were measured every 2 days. (I) Representative images of immunohistochemistry (IHC) staining for HDAC2, NLRP3, H3K27ac and p‐P65 in clinical CRC specimens. Scale bar: 50 µm. Correlations (Fisher's exact test) between HDAC2 IHC scores and IHC scores of three proteins (NLRP3, H3K27ac, p‐P65) in CRC tissues were displayed for our cohort (*n* = 82). (J) Analysis of overall survival of CRC patients in our institution categorised by HDAC2 and NLRP3 status using Kaplan‒Meier survival curves, *n* = 200, statistical significance was evaluated by the log‐rank test. Statistical significance is indicated (^*^ *p* < .05, ^**^ *p* < .01, ^***^ *p* < .001, ^****^ *p* < .0001).

In vivo, the impact of combined anticancer drugs and the HDAC2 inhibitor SCA on CRC progression was further evaluated using the colorectal PDX model. Following the generation of CRC PDX models as detailed in Section 2 (Figure 7A), two PDX models (BP0036 and BP0159) confirmed to have high HDAC2 expression were identified for further evaluation (Figure S11A). Analysis of tumour lysates using Western blotting showed elevated Cleaved GSDMD, indicative of pyroptosis, in the combination therapy group but not in the single‐agent groups for both PDX models (Figures 7B and S11B). The tumour size was significantly reduced in the regorafenib plus SCA group compared to the control group. However, neither regorafenib nor SCA alone showed significant suppression of tumour growth in vivo (Figures 7C,D and S11C,D). Concurrent treatment with 5‐FU and SCA blockade showed similar results. Afterwards, we then assessed the response of the PDX models to anticancer drugs by measuring the tumour growth inhibition rate. The results showed that after 12 days of treatment with the same drug doses, the tumour growth inhibition rate was notably greater in the regorafenib plus SCA group compared to the group treated with regorafenib alone in the BP0036 model (inhibition rate 91.11 ± 3.408% vs. 37.84 ± 6.793%) (Figure 7E). Similarly, the inhibition rate in the 5‐FU plus SCA group was much higher than that in the 5‐FU alone group (inhibition rate 93.42 ± 1.399% vs. 42.65 ± 8.009%). The same trend was observed for Model BP0159 (Figure S11E). We also validated these findings in SW620 colon cancer xenografts, where combination therapy conferred superior tumour inhibition over monotherapy (Figure 7F‒H). Moreover, compared to 5‐FU and oxaliplatin alone, combining the HDAC2‐selective inhibitor with the regimen resulted in significantly reduced tumour burden (Figure S11F). And an increase in the expression of NLRP3 and cleaved GSDMD is observed in the combined SCA treatment group (Figure S11G). To rigorously evaluate the specificity of SCA, we conducted rescue experiments utilising RNA interference (RNAi) to selectively deplete NLRP3. Notably, siRNA‐mediated NLRP3 knockdown potently rescued pyroptosis induced by HDAC2 inhibitor treatment, abrogating GSDMD cleavage and LDH release (Figure S12A,B). In addition, NLRP3 knockdown reversed cytotoxic effects and pyroptosis rate induced by SCA (Figure S12C,D). These findings confirm that the pyroptotic response induced by SCA is critically dependent on NLRP3. These results demonstrate that the combination of an HDAC2 inhibitor (SCA) can enhance CRC sensitivity to antitumour therapies.

**FIGURE 8:**
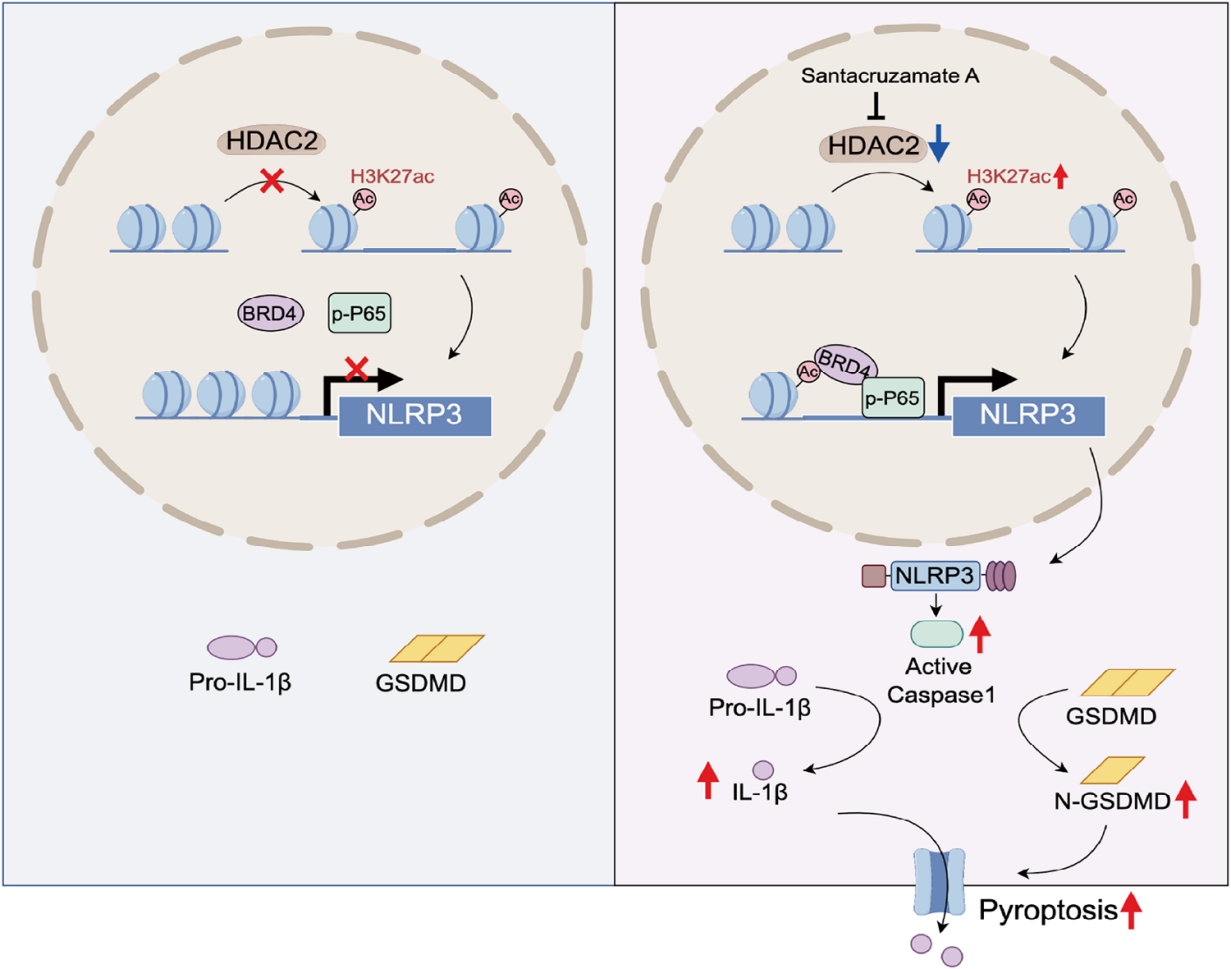
Schematic illustrating inhibition of HDAC2 can reverse the epigenetic silencing of NLRP3 to induce pyroptosis in colorectal cancer.

Previous studies have shown that HDAC2 overexpression correlates with poor prognosis in CRC patients.^25^, ^26^ To delve deeper into the clinical relevance of our results, we conducted immunohistochemical staining to analyse the expression levels of HDAC2, NLRP3, H3K27ac and p‐P65 in a cohort consisting of 82 CRC patient specimens (Figure 7I). We observed an inverse correlation between the expression of HDAC2 and the levels of p‐P65, H3K27ac and NLRP3. Statistical analysis verified the significance of these negative correlations. Moreover, we included patients with rectal cancer who received neoadjuvant therapy and stratified them according to pretreatment HDAC2 expression levels to assess treatment response. Patients exhibiting low expression levels of HDAC2 demonstrated increased treatment sensitivity as evidenced by magnetic resonance imaging compared to those with high HDAC2 expression (Figure S13A). Kaplan‒Meier survival analysis indicated that patients with high HDAC2 expression experienced shorter overall survival (Figure 7J). Moreover, further analysis revealed that patients with high HDAC2 and low NLRP3 expression had a poorer prognosis. Similar overall survival patterns were observed in patients within the GSE database through analysis (Figure S13B). These results demonstrate that the correlative expression of HDAC2 and NLRP3 serves as a prognostic biomarker for patients with CRC. In conclusion, all results provided an illustration of a working model through which KO of HDAC2 permits NLRP3 re‐expression, subsequently initiating pyroptotic signalling (Figure 8).

## 4 DISCUSSION

CRC is the third most common malignancy worldwide.^27^ Although chemotherapy and targeted therapy are the main interventions, the survival results are unsatisfactory.^3^ Accumulating evidence indicates that triggering pyroptosis could significantly improve tumour treatment response.^8^, ^9^ Although the pyroptotic executioner protein GSDMD has been extensively studied, whether GSDMD‐dependent pyroptosis can be activated in CRCs remains ambiguous. In this study, we identified a novel HDAC2‒NLRP3 pathway that governs GSDMD‐mediated pyroptosis in CRC. The primary aim of the research was to elucidate the potential of HDAC2‐mediated pyroptosis in sensitising CRC cells to various antitumour drugs, including chemotherapeutics and targeted therapies. Therefore, we selected 5‐FU and regorafenib as representative drugs for our analysis. High expression of HDAC2 in CRC induces chromatin condensation and transcriptional suppression at the NLRP3 promoter. Consequently, CRC cells cannot undergo pyroptosis following regorafenib treatment. In contrast, HDAC2 knockdown increased H3K27 acetylation in the NLRP3 promoter region, enhancing chromatin accessibility. Concurrently, the NF‐κB signalling cascade is activated in CRC cells under regorafenib treatment, with increased translocation and phosphorylation of the P65 transcription factor. Elevated H3K27ac recruits more p‐P65 through BRD4, promoting NLRP3 transcription and thus restoring NLRP3 expression. NLRP3 assembles into an inflammasome complex that activates Caspase1, which cleaves GSDMD and Pro IL‐1β to trigger pyroptosis. Additionally, the therapeutic response in CRC xenografts and PDXs was dramatically improved using the HDAC2 inhibitor, SCA. Elevated levels of HDAC2 and decreased levels of NLRP3 are associated with unfavourable outcomes in individuals diagnosed with CRC.

Emerging evidence has revealed that various anticancer therapies can elicit GSDMD‐dependent tumour cell pyroptosis by activating the NLRP3 inflammasome, including chemotherapy and targeted therapy. Yan et al. found that cisplatin triggers pyroptosis through the NLRP3/Caspase1/GSDMD pathway in triple‐negative breast cancer.^28^ Two chemotherapeutics (doxorubicin and cisplatin) can activate the NLRP3‒GSDMD pyroptosis axis to exert antitumour effects in malignant mesothelioma.^29^ Furthermore, targeted therapies have demonstrated the ability to initiate pyroptosis within cancerous cells. Activation of the Caspase1/GSDMD pathway by Val‐boroPro leads to pyroptosis in acute myeloid leukaemia.^30^ In CRC, emerging drugs have been reported to upregulate NLRP3 expression and activate the NLRP3 inflammasome, leading to pyroptosis.^31^, ^32^ Although these drugs have the potential to induce pyroptosis, they are not specific and therefore still show potential for the development of more targeted pyroptosis‐inducing drugs. A recent study showed significant alterations in the expression of NLRP3 inflammasome component genes in 15 of the 24 cancer types.^33^ Hence, we investigated the levels of essential molecules involved in the NLRP3‒GSDMD pyroptosis pathway in CRC, as well as the potential for inducing this pathway with certain agents. These findings showed that GSDMD is ubiquitously expressed in various CRC cells. Surprisingly, GSDMD‐induced pyroptosis following drug administration has rarely been observed in colorectal carcinoma cell lines. Further research has confirmed that the silencing of NLRP3 expression in most CRC cell lines is the primary reason for the blockage of this pathway. Therefore, reversing the silencing of NLRP3 could be crucial for inducing pyroptosis in CRC.

Earlier research has shown that NLRP3 expression can be modulated through various mechanisms such as ribosomal stalling, translational inhibition and post‐translational modifications.^34^ The ubiquitination and phosphorylation of NLRP3 has been extensively investigated.^35^ Here, we found that NLRP3 mRNA was downregulated in both TCGA data and CRC cell lines, indicating that transcriptional or pretranscriptional regulation is the key to NLRP3 silencing in CRC. Silencing gene expression via epigenetic repression is both stable and durable. Currently, some evidence suggests that epigenetic modifications may be involved in the regulation of NLRP3 inflammasome expression and activation. For example, in lung cancer, LINC00969 modulates H3K27me3 levels at the NLRP3 promoter region and post‐transcriptionally modifies NLRP3 m6A levels in an m6A‐YTHDF2‐dependent manner, thereby epigenetically suppressing NLRP3.^35^ Studies have confirmed that HDAC2, which belongs to the HDAC family, is overexpressed in CRC.^36^, ^37^ Our results demonstrate that aberrant hyperexpression of HDAC2 is the key driver of epigenetic silencing of NLRP3 in CRC. Previous studies have confirmed that APC loss in CRC trigger aberrant activation of the Wnt pathway, resulting in heightened expression of HDAC2. In addition, the transcription factor c‐Myc is involved in mediating HDAC2 transcriptional upregulation in response to Wnt pathway activation. The above conclusions were verified in this study. Additionally, HDAC2 KO enhanced H3K27 acetylation at the NLRP3 promoter region, leading to a relaxed chromatin structure and facilitation of NLRP3 transcription.

Inhibition of HDAC2 leads to elevated histone acetylation at gene promoters.^38^ Our results revealed that acetylation of H3K27, but not H3K9, was enriched in the NLRP3 promoter upon HDAC2 KO. H3K27ac is an active epigenetic marker that modulates gene transcription.^39^ The bromodomain‐containing protein BRD4 recognises and directly engages acetylated H3K27, thereby serving as a molecular scaffold that facilitates the recruitment of transcription factors to chromatin.^40^ Additionally, multiple studies have shown that BRD4 interacts with NF‐κB p65 to activate transcription.^41^, ^42^ Therefore, we hypothesised that the H3K27ac reader BRD4 may scaffold the interaction between the NLRP3 promoter and transcription factor p65 to drive NLRP3 expression. Our data revealed that H3K27ac forms a complex with phosphorylated P65 in a BRD4‐dependent manner. Moreover, p‐P65 occupancy at the NLRP3 promoter and subsequent NLRP3 transcription were reduced upon BRD4 knockdown, confirming BRD4's role as a scaffold between p‐P65‐ and H3K27ac‐enriched chromatin. In summary, our results elucidate a novel mechanism whereby HDAC2 inhibition leads to increased H3K27 acetylation at the NLRP3 promoter, which is recognised by BRD4 and enables p‐P65 recruitment to re‐express the NLRP3 gene.

This study demonstrated that targeting HDAC2 can enhance the efficacy of antitumour drug treatment in CRC. Growing evidence suggests that HDAC2 is elevated in colorectal tumours and is associated with adverse clinical outcomes.^43^, ^44^ We verified that HDAC2 is amplified in CRC, its expression is negatively correlated with NLRP3, and combined analysis of HDAC2 and NLRP3 can be used as an indicator to predict patient survival. In the last 10 years, Preclinical and clinical studies have indicated the potential of HDAC inhibitors as effective anticancer drugs.^45^, ^46^ Specifically, these inhibitors have received approval for treating various hematopoietic cancers, such as cutaneous T‐cell lymphomas.^47^ Our findings support the utility of HDAC2 inhibitors in colorectal treatment. SCA, a powerful and specific HDAC2 inhibitor, has been demonstrated to effectively suppresses tumour progression both in vitro and in vivo.^48^, ^49^ In our study, aberrant HDAC2 overexpression led to the transcriptional silencing of NLRP3 and suppressed cancer cell pyroptosis. Targeting HDAC2 with the small‐molecule inhibitor SCA increased the efficacy of anticancer drugs in xenografts and PDX models. Thus, epigenetic modulation of pyroptosis by HDAC inhibitors may provide important opportunities for enhancing antitumour efficacy.

## 5 CONCLUSION

In conclusion, our results emphasise that the excessive expression of HDAC2 in CRC leads to the epigenetic suppression of NLRP3, impeding pyroptosis induced by drug treatments. Inhibition of HDAC2 facilitates the formation of the H3K27ac‒BRD4‒p‐P65 complex, thereby promoting NLRP3 transcription. Consequently, targeting HDAC2 has emerged as a new approach to enhance the effectiveness of conventional therapeutic modalities.

## AUTHOR CONTRIBUTIONS

Xin Guan, Chao Liu, Yuanfei Yao and Yanqiao Zhang contributed to the study conception and design. Xin Guan, Ruiqi Liu, Bojun Wang, Ruxin Xiong and Luying Cui performed in vitro experiments with the cell lines. Xin Guan, Bojun Wang and Yuanyu Liao provided expertise in murine models. Yuli Ruan performed TCGA dataset analysis. Lin Fang, Xiaolin Lu and Xuefan Yu gathered data from patients with CRC, while Dan Su, Yue Ma, Zhuo Chen and Tianjiao Dang conducted subsequent monitoring. Xin Guan, Chao Liu, Yuanfei Yao and Yanqiao Zhang wrote and revised the manuscript. All authors have read and approved the final manuscript.

## CONFLICT OF INTEREST STATEMENT

The authors declare they have no conflicts of interest.

## ETHICS STATEMENT

The study was approved by the Ethics Committee of Harbin Medical University (no. KY2022‐20). Each participant provided written consent, authorising the utilisation of their biological samples and personal data for research objectives. All animal procedures were approved by the Institutional Animal Care and Use Committee of Harbin Medical University (Harbin, China), and the experiments strictly adhered to the tumour induction guidelines for mice.

## Supporting information

Supporting information

Supporting information

Supporting information

Supporting information

Supporting information

Supporting information

Supporting information

Supporting information

Supporting information

Supporting information

Supporting information

Supporting information

Supporting information

Supporting information

Supporting information

Supporting information

Supporting information

## Data Availability

All data generated or analysed during this study are included in this published article and its supplementary information files. Gene expression data with standard annotations were downloaded from The Cancer Genome Atlas and GTEx. GSE17536 data were downloaded from the GEO datasets. The data generated in this study are publicly available under accession code HRA005317. The data that support the findings of this study are openly available in the Genome Sequence Archive at the website https://ngdc.cncb.ac.cn/gsa/.
