# Supplementary material for: Inhibition of HDAC2 sensitises antitumour therapy by promoting NLRP3/GSDMD‐mediated pyroptosis in colorectal cancer
Source: Clin Transl Med. 2024 May 28;14(6):e1692. doi: 10.1002/ctm2.1692 (PMC11131357; doi:10.1002/ctm2.1692)
Supplement: Supplementary file 1 — Supporting information [file CTM2-14-e1692-s014.docx]

**
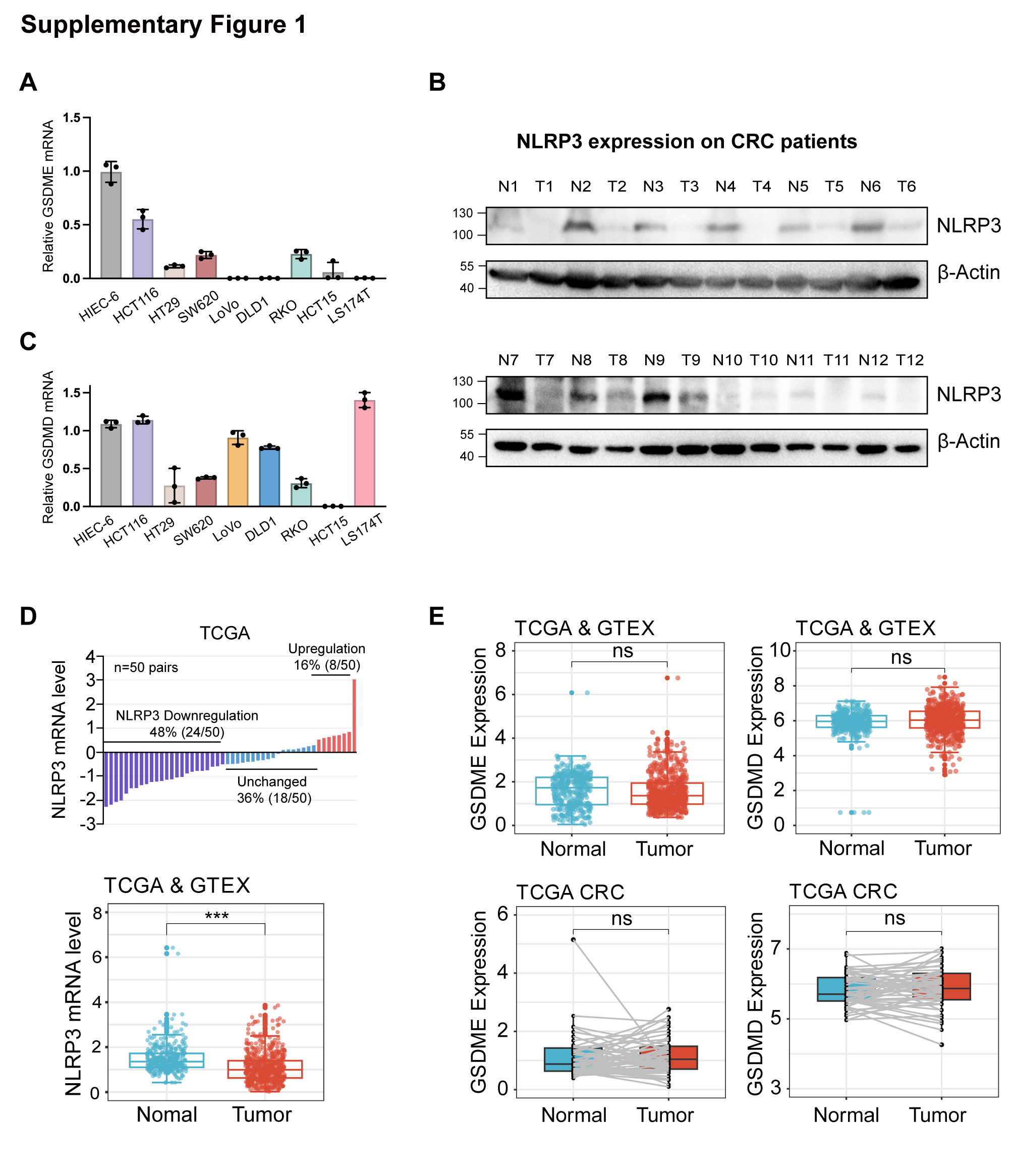
**

**Fig. S1 NLRP3 expression is silenced in colorectal cancer. A** GSDME mRNA levels were evaluated using qPCR and compared between human CRC cells and HIEC-6 cells. **B** Western blot analysis of NLRP3 protein levels in 12 matched pairs of colorectal cancer tissues and neighboring normal tissues. **C** GSDMD mRNA levels were evaluated using qPCR and compared between human CRC cells and HIEC-6 cells. **D** NLRP3 mRNA levels were analyzed in 50 matched pairs of CRC tissues and adjacent normal tissues using TCGA data. FC: fold change (tumor/normal) (upper panel). A joint analysis of NLRP3 expression in CRC cancer tissues and their corresponding normal tissues was conducted using data from the TCGA and GTEx databases (lower panel). **E** A joint analysis of GSDMD or GSDME expression in CRC cancer tissues and their corresponding normal tissues was conducted using data from the TCGA and GTEx databases (upper panel). GSDMD or GSDME mRNA levels were analyzed in 50 matched pairs of CRC tissues and adjacent normal tissues, using TCGA data (lower panel).
