# Supplementary material for: Inhibition of HDAC2 sensitises antitumour therapy by promoting NLRP3/GSDMD‐mediated pyroptosis in colorectal cancer
Source: Clin Transl Med. 2024 May 28;14(6):e1692. doi: 10.1002/ctm2.1692 (PMC11131357; doi:10.1002/ctm2.1692)
Supplement: Supplementary file 2 — Supporting information [file CTM2-14-e1692-s002.docx]

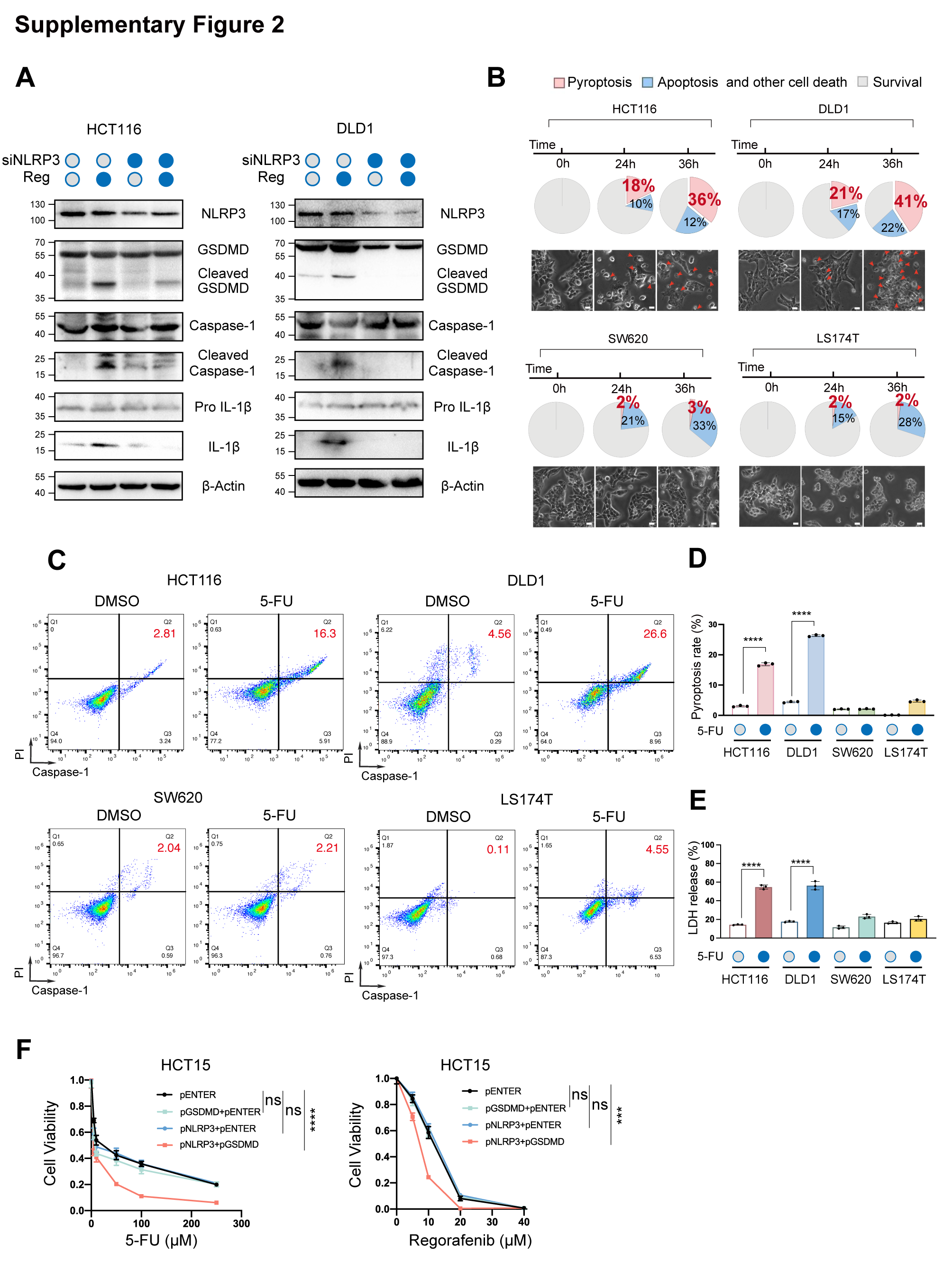


**Fig. S2 Silencing of NLRP3 in CRC attenuates GSDMD-mediated pyroptosis induced by anticancer drugs. A** CRC cells transfected with siNLRP3 and control cells were treated with 10 μM regorafenib (Reg) for 24 hours. Pyroptosis-related proteins were assessed by Western blot analysis. **B** Cell death induced by 5-FU in CRC cancer cells was morphologically assessed via microscopy. Red arrowheads indicate large bubbles and pores emerging from the plasma membrane. Dead cells were tallied and quantified based on five separate images. Scale bar: 50 μm. Statistical significance is indicated. **C,D** Flow cytometry analysis for activated Caspase-1/PI. **E** CRC cells were evaluated for pyroptosis 24 hours posttreatment with 5-FU, as assessed by LDH release. **F** CCK-8 assays were performed to detect the viability of HCT15 cells transfected with different plasmids after treatment with varying concentrations of regorafenib for 48 h.
