# Supplementary material for: Inhibition of HDAC2 sensitises antitumour therapy by promoting NLRP3/GSDMD‐mediated pyroptosis in colorectal cancer
Source: Clin Transl Med. 2024 May 28;14(6):e1692. doi: 10.1002/ctm2.1692 (PMC11131357; doi:10.1002/ctm2.1692)
Supplement: Supplementary file 3 — Supporting information [file CTM2-14-e1692-s012.docx]

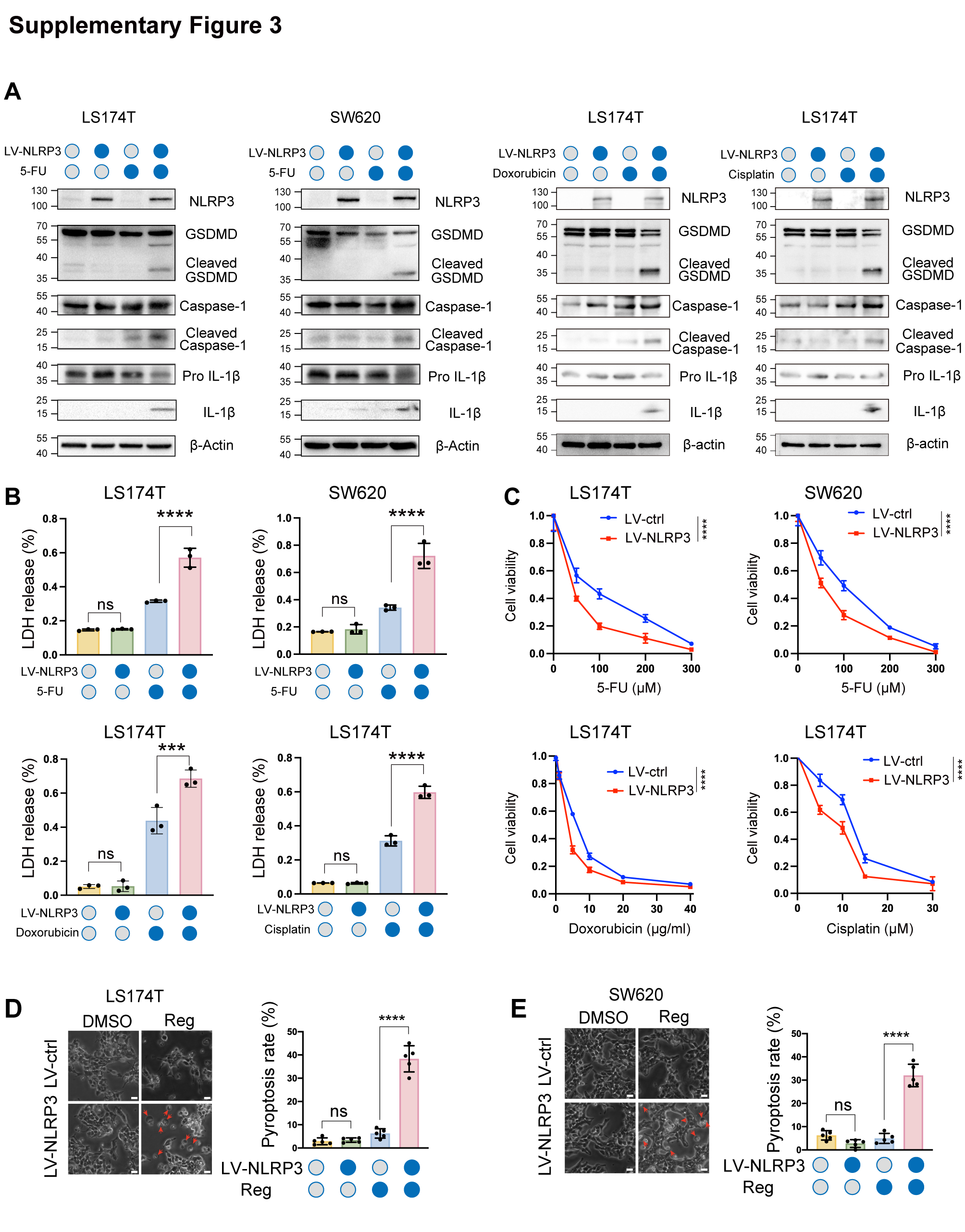


**Fig. S3 Supplementation of NLRP3 expression in CRC cells rescues GSDMD-mediated pyroptosis under antitumor drugs. A-C** Cells stably expressing NLRP3 and control cells were treated with various chemotherapeutic drugs for 24 hours. Pyroptosis-related proteins were assessed by Western blot analysis (A). LDH release was measured to evaluate pyroptosis levels (B). CCK8 Assay is used to detect cell activity (C). **D, E** Typical bright-field microscopic images of LS174T and SW620 cells are shown. Large bubbles protruding from the plasma membrane are highlighted by red arrows. Scale bar: 50 μm. Dead cells were tallied and quantified based on five separate images.
