# Supplementary material for: Inhibition of HDAC2 sensitises antitumour therapy by promoting NLRP3/GSDMD‐mediated pyroptosis in colorectal cancer
Source: Clin Transl Med. 2024 May 28;14(6):e1692. doi: 10.1002/ctm2.1692 (PMC11131357; doi:10.1002/ctm2.1692)
Supplement: Supplementary file 4 — Supporting information [file CTM2-14-e1692-s006.docx]

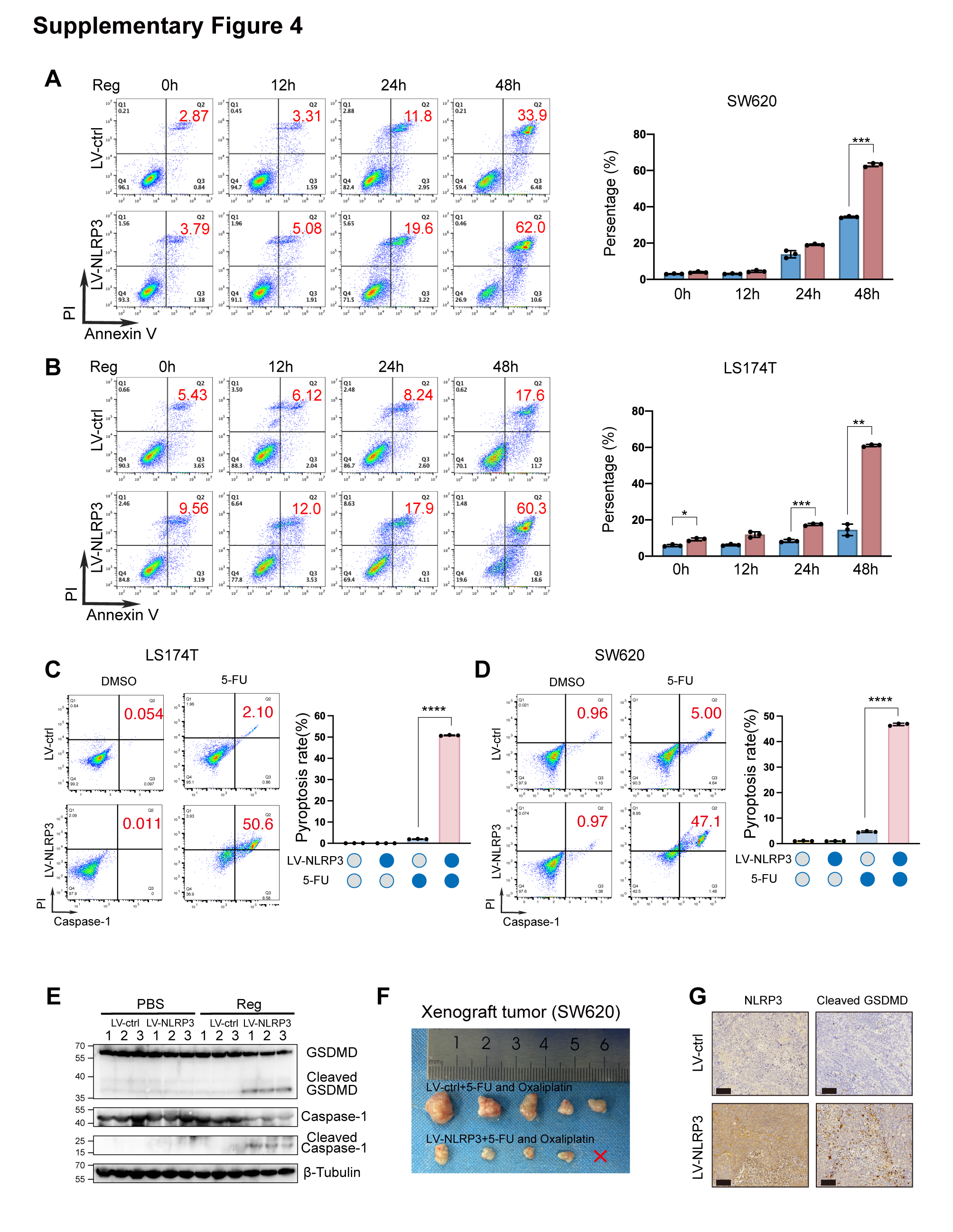


**Fig. S4 Restoring NLRP3 expression in colorectal cancer re-induced pyroptosis in vivo and in vitro. A, B** The percentage of Annexin V FITC and Propidium Iodide (PI) double-positive cells in LS174T or SW620 cells were treated with 10 μM regorafenib at the indicated time points. Double-positive cells in LS174T and SW620 were detected by flow cytometry in each treatment group. **C,** **D** Flow cytometry analysis for activated Caspase-1/PI. **E** Expression of Cleaved Caspase-1 and Cleaved GSDMD in tumor tissues was analyzed by Western blot. **F** SW620 xenografts were established in nude mice and treated as shown (n=5/group). **G** The expression of NLRP3 and cleaved GSDMD in xenografts was analyzed by IHC. Scale bar: 100 μm.
