# Supplementary material for: Inhibition of HDAC2 sensitises antitumour therapy by promoting NLRP3/GSDMD‐mediated pyroptosis in colorectal cancer
Source: Clin Transl Med. 2024 May 28;14(6):e1692. doi: 10.1002/ctm2.1692 (PMC11131357; doi:10.1002/ctm2.1692)
Supplement: Supplementary file 5 — Supporting information [file CTM2-14-e1692-s016.docx]

**
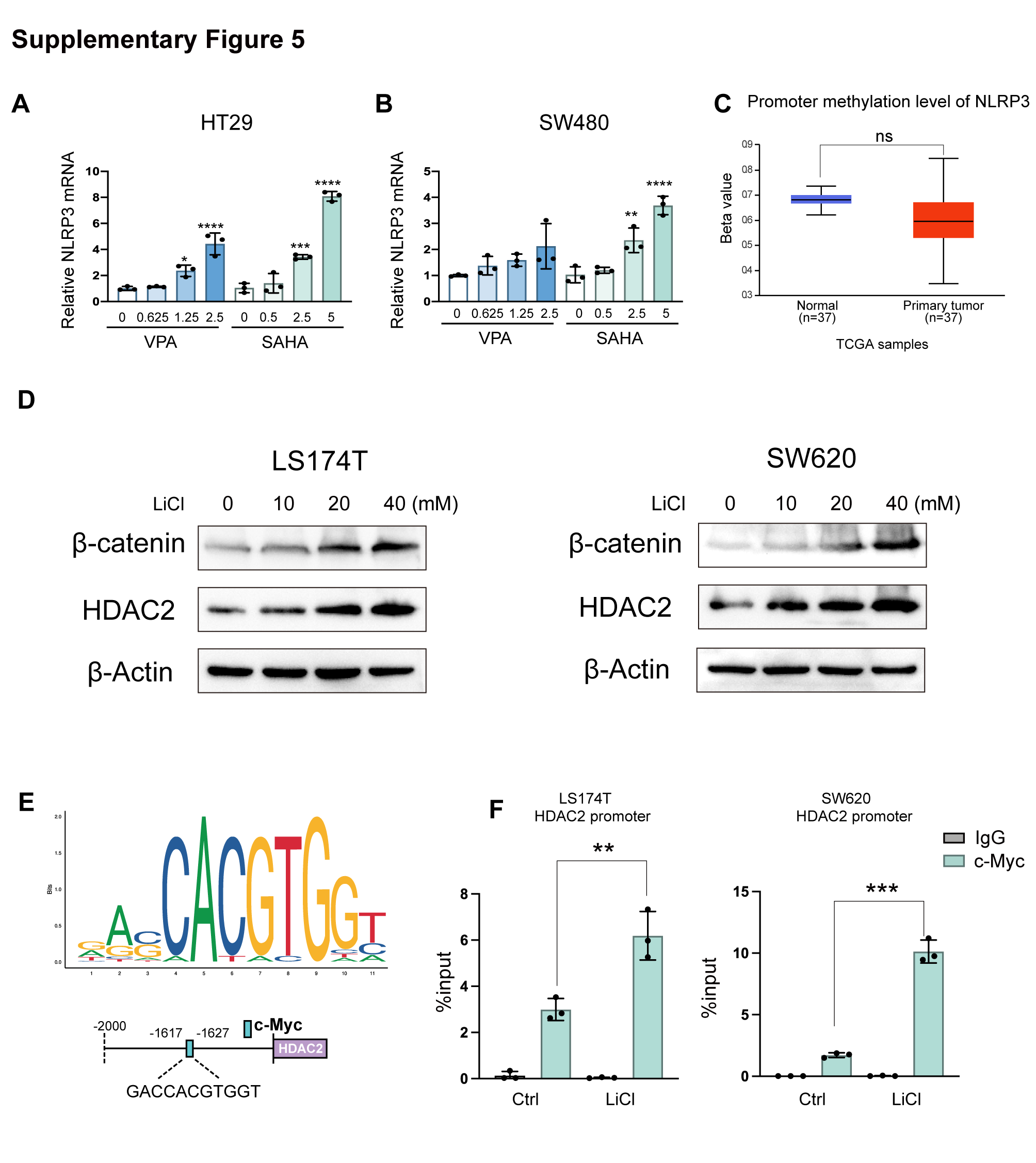
**

**Fig. S5 Abnormal activation of the Wnt pathway in CRC leads to increased HDAC2 expression. A, B** RT-qPCR analysis of NLRP3 transcript levels in HT29 and SW480 cells following exposure to increasing doses of HDAC pan-inhibitors. Two distinct small molecule HDAC inhibitors were utilized independently. **C** Methylation analysis of the NLRP3 promoter region was performed using colorectal adenocarcinoma data from TCGA. The Beta value indicates the level of DNA methylation ranging from 0 (unmethylated) to 1 (fully methylated). **D** Western blot analyses were conducted to assess the expression levels of HDAC2 and β-catenin in LS174T and SW620 cells after exposure to escalating doses of LiCl. **E** Schematic representation illustrating the positions of potential c-Myc binding sites in the promoter region of the HDAC2 gene. **F** ChIP-qPCR validated the interaction between c-Myc and the HDAC2 promoter in LS174T and SW620 cells under conditions with or without LiCl.
