# Supplementary material for: Inhibition of HDAC2 sensitises antitumour therapy by promoting NLRP3/GSDMD‐mediated pyroptosis in colorectal cancer
Source: Clin Transl Med. 2024 May 28;14(6):e1692. doi: 10.1002/ctm2.1692 (PMC11131357; doi:10.1002/ctm2.1692)
Supplement: Supplementary file 6 — Supporting information [file CTM2-14-e1692-s003.docx]

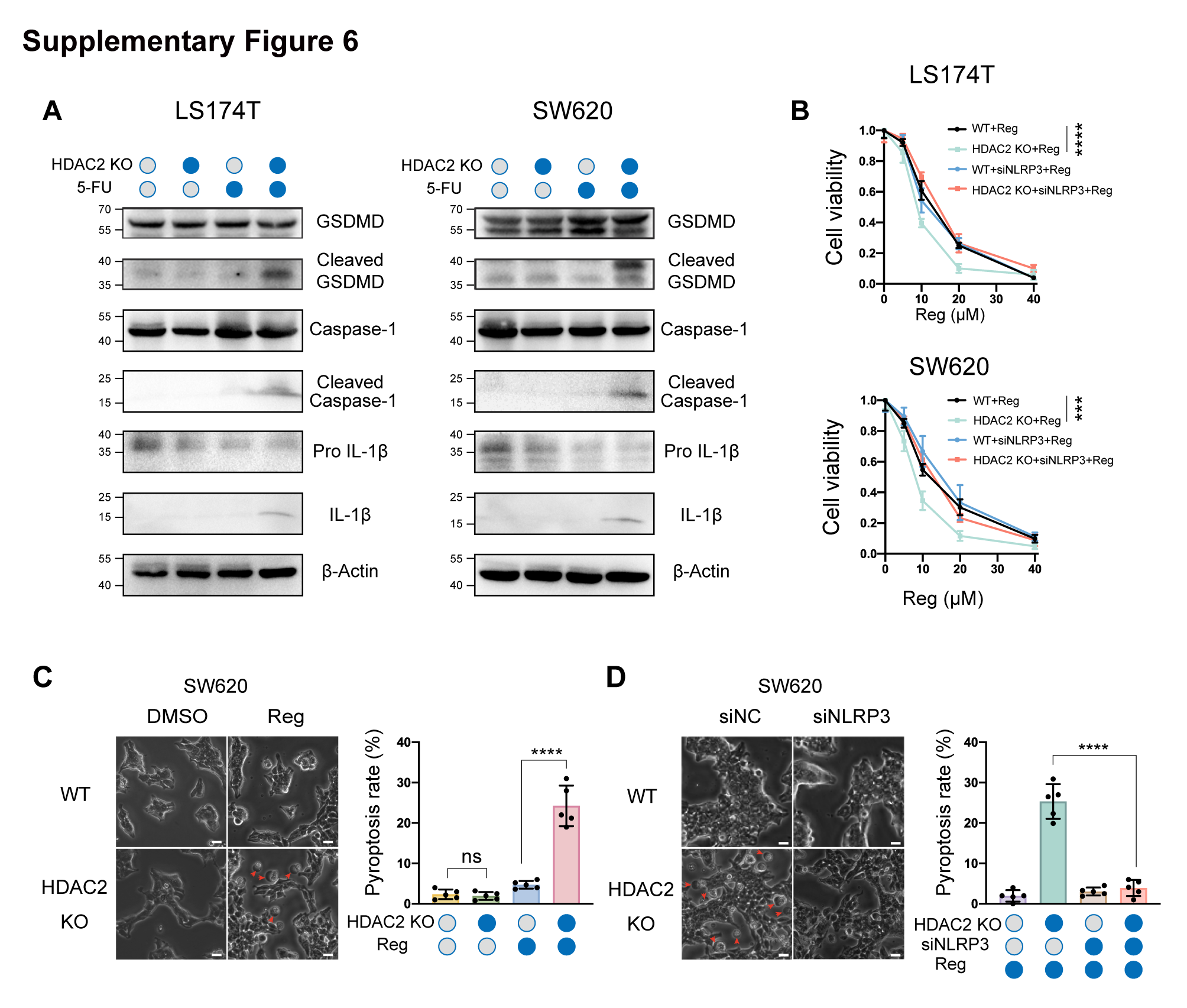


**Fig. S6 Downregulating HDAC2 can induce GSDMD-pyroptosis by the restoration of NLRP3. A** LS174T and SW620 HDAC2 knockout lines were generated with CRISPR/Cas9, and treated with 25 μM 5-FU. Pyroptosis pathway proteins were analyzed by western blot. **B** CCK-8 assay was utilized to analyze cell viability across various treatment groups. **C, D** SW620 HDAC2 knockout lines were treated with 10 μM regorafenib, and transfected with NLRP3 siRNA for rescue experiments. Typical bright-field microscopic images of SW620 cells are shown. Large bubbles protruding from the plasma membrane are highlighted by red arrows. Scale bar: 50 μm. The displayed data represent the mean ± SD from three separate experiments.
