# Supplementary material for: Inhibition of HDAC2 sensitises antitumour therapy by promoting NLRP3/GSDMD‐mediated pyroptosis in colorectal cancer
Source: Clin Transl Med. 2024 May 28;14(6):e1692. doi: 10.1002/ctm2.1692 (PMC11131357; doi:10.1002/ctm2.1692)
Supplement: Supplementary file 7 — Supporting information [file CTM2-14-e1692-s011.docx]

**
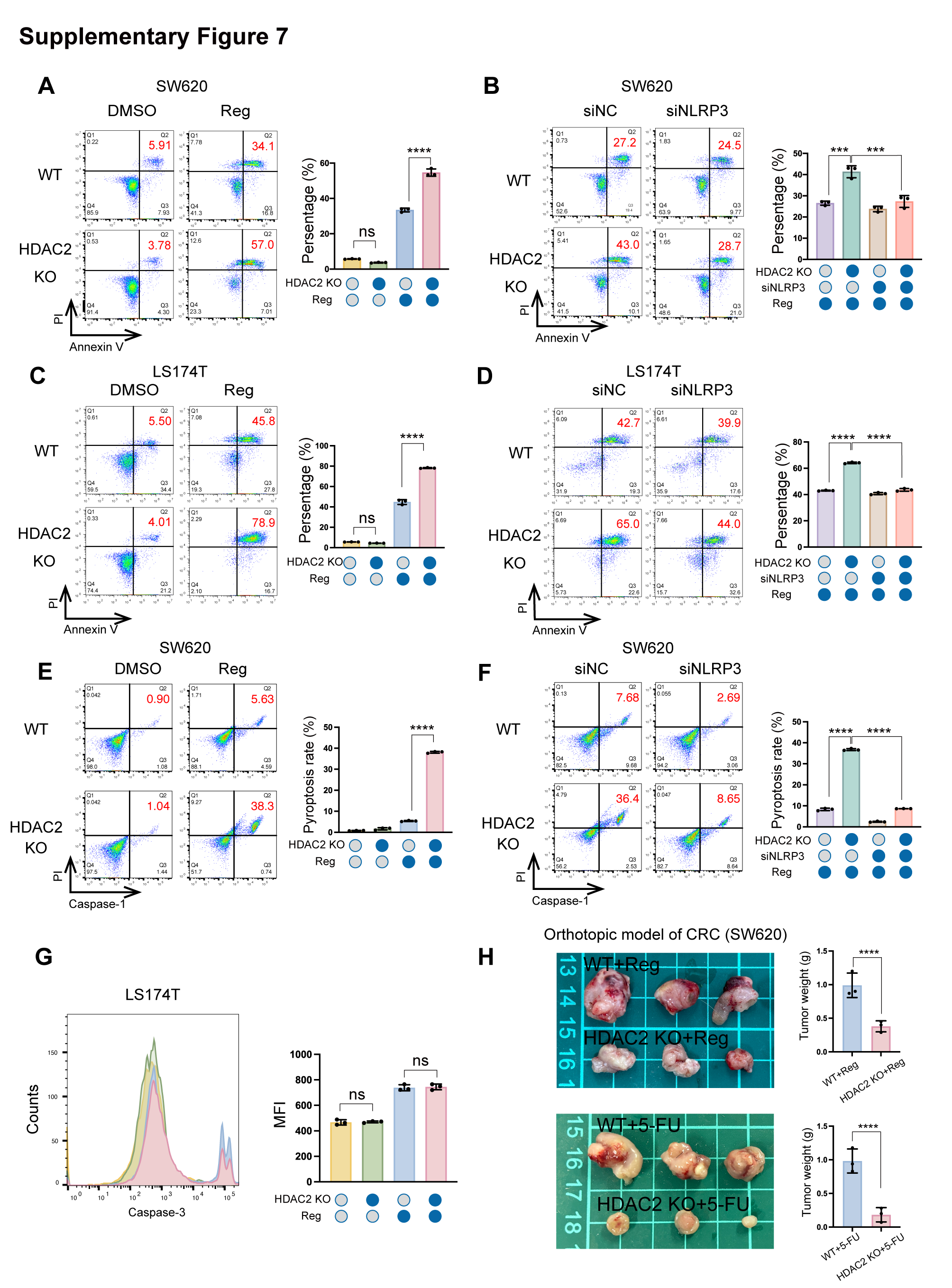
**

**Fig. S7** **Knocking out HDAC2 activates GSDMD-mediated pyroptosis by upregulating NLRP3.** **A-D** The percentage of Annexin V and Propidium Iodide (PI) double-positive cells in CRC cells were detected by flow cytometry in each treatment group. **E, F** Flow cytometry analysis for activated Caspase-1/PI. **G** Flow cytometry analysis for activated Caspase-3. Data are expressed as the mean of the fluorescence intensity (MFI). **H** Tumor weight in orthotopic colon cancer mouse model treated with regorafenib or 5-FU. Statistical significance is indicated (*p < 0.05, **p < 0.01, ***p < 0.001, ****p < 0.0001).
