# Supplementary material for: Inhibition of HDAC2 sensitises antitumour therapy by promoting NLRP3/GSDMD‐mediated pyroptosis in colorectal cancer
Source: Clin Transl Med. 2024 May 28;14(6):e1692. doi: 10.1002/ctm2.1692 (PMC11131357; doi:10.1002/ctm2.1692)
Supplement: Supplementary file 8 — Supporting information [file CTM2-14-e1692-s007.docx]

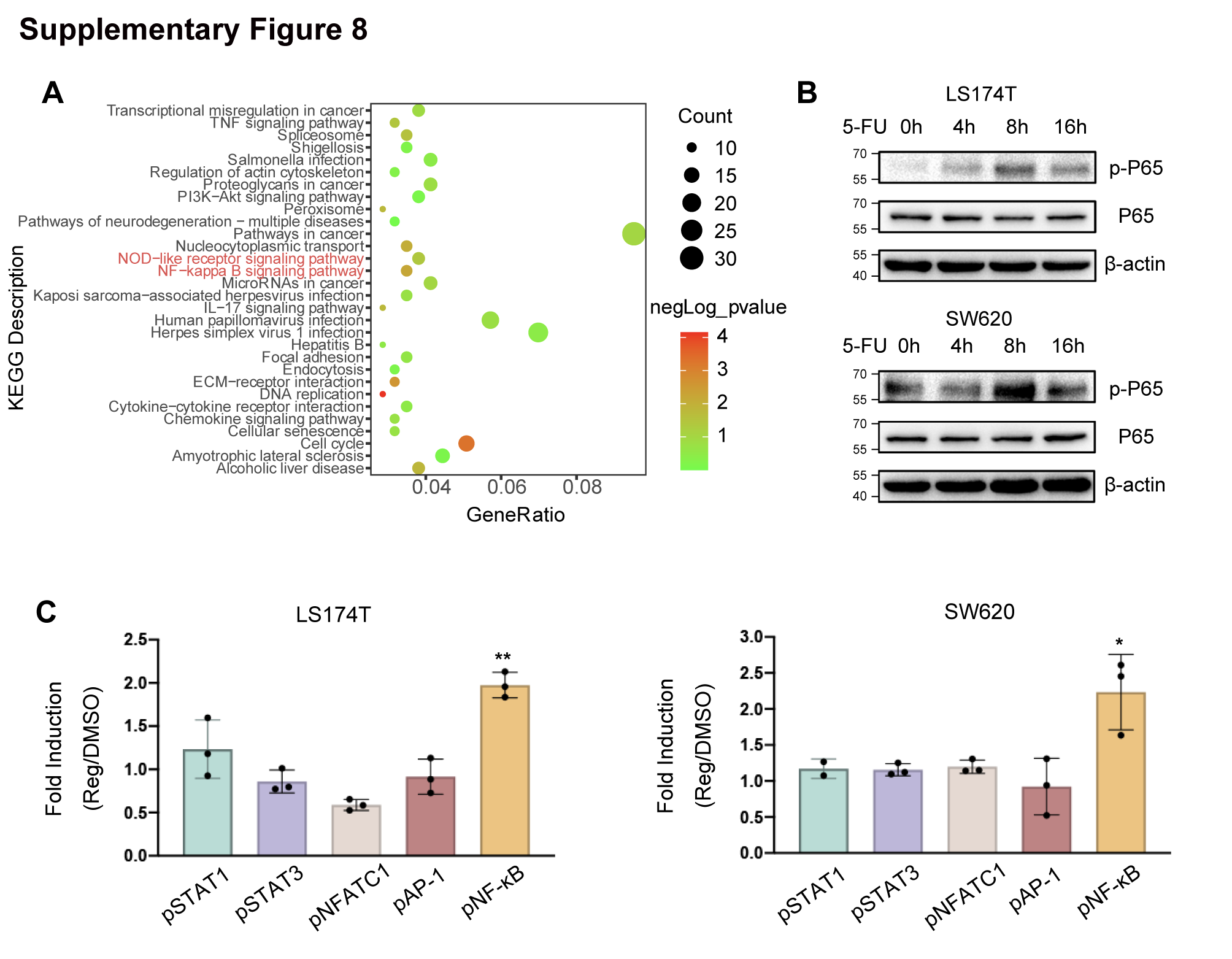


**Fig. S8 NF-κB Signaling can be activated by HDAC2 knockout and anti-tumor drug treatment. A** Differentially expressed genes were identified by integrating ATAC-Seq and RNA-Seq data, and KEGG pathway analysis revealed significant enrichment among these genes. **B** The levels of total total P65 and p-P65 were analyzed by Western blot in LS174T or SW620 cells treated with 25 μM 5-FU at the indicated time points. **C** LS174T and SW620 cells were treated with 10 μM regorafenib and transcriptional activity of multiple transcription factors was detected using a dual-luciferase reporter assay.
