# Supplementary material for: Inhibition of HDAC2 sensitises antitumour therapy by promoting NLRP3/GSDMD‐mediated pyroptosis in colorectal cancer
Source: Clin Transl Med. 2024 May 28;14(6):e1692. doi: 10.1002/ctm2.1692 (PMC11131357; doi:10.1002/ctm2.1692)
Supplement: Supplementary file 9 — Supporting information [file CTM2-14-e1692-s008.docx]

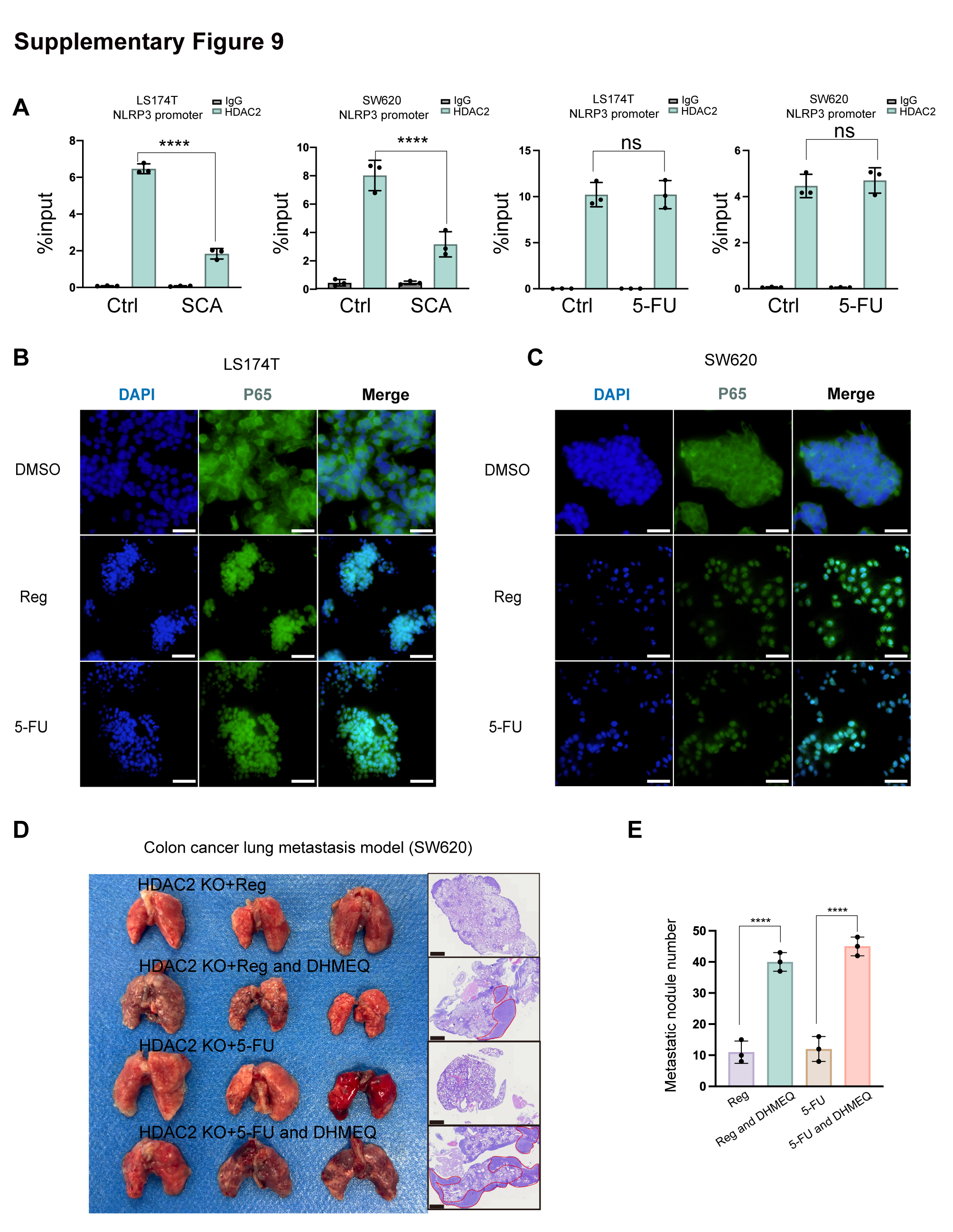


**Fig. S9 Absence of HDAC2 enhances NLRP3 promoter chromatin accessibility, recruiting more p-P65. A** ChIP-PCR validated the interaction between HDAC2 and the NLRP3 promoter in LS174T and SW620 cells under conditions with or without SCA treatment. The above experiments were performed under the conditions of 5-FU treatment. **B, C** The subcellular localization of P65 was assessed by immunofluorescence staining using P65 antibodies. Images were acquired using a confocal microscope. Scale bar: 50 μm. **D** The lung metastases were visualized under different treatment regimens and identified by hematoxylin and eosin (H&E) staining. **E** The number of lung metastasis nodules in different treatment groups. Statistical significance is indicated (*p < 0.05, **p < 0.01, ***p < 0.001, ****p < 0.0001).
