# Supplementary material for: Inhibition of HDAC2 sensitises antitumour therapy by promoting NLRP3/GSDMD‐mediated pyroptosis in colorectal cancer
Source: Clin Transl Med. 2024 May 28;14(6):e1692. doi: 10.1002/ctm2.1692 (PMC11131357; doi:10.1002/ctm2.1692)
Supplement: Supplementary file 10 — Supporting information [file CTM2-14-e1692-s017.docx]

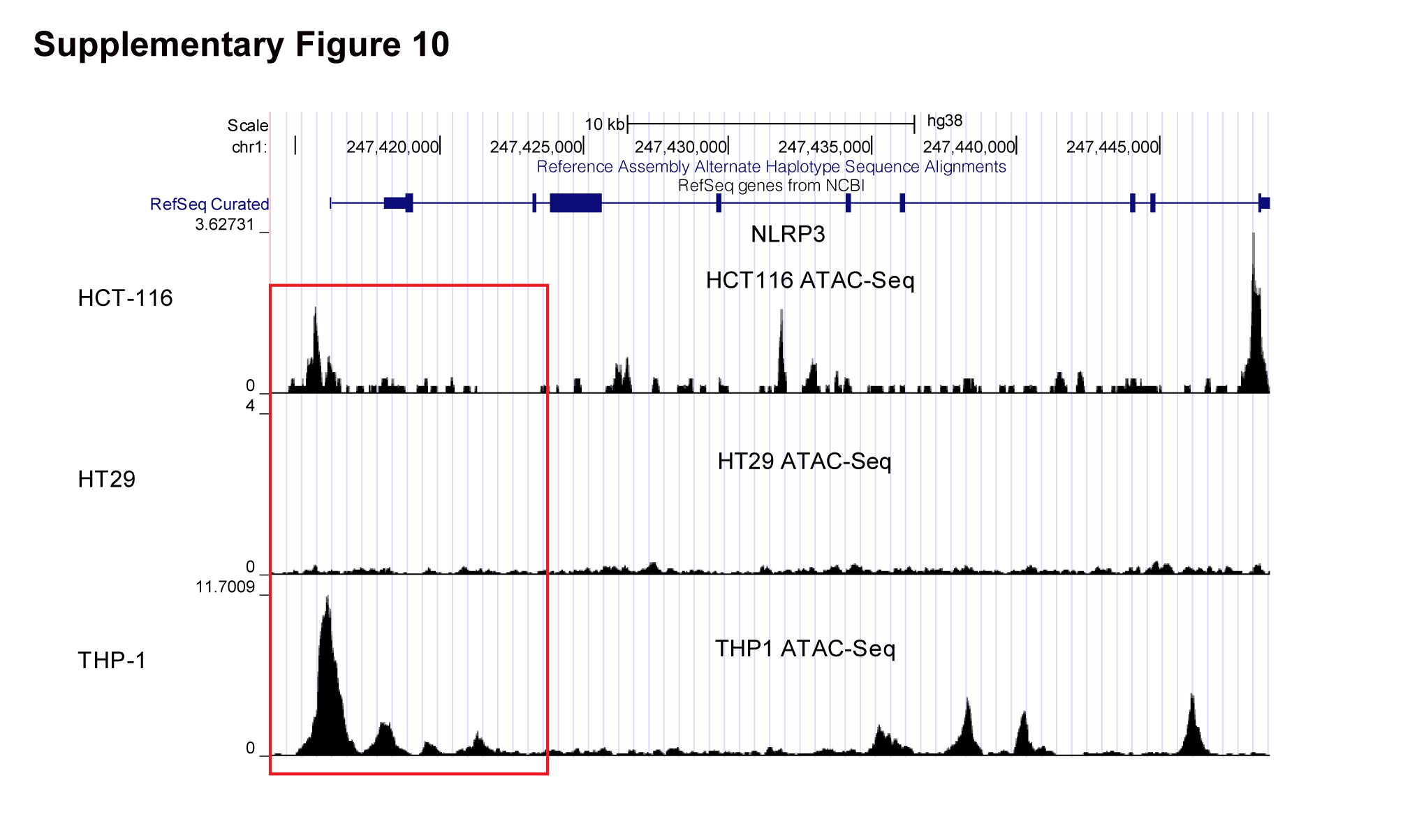


**Fig. S10 ATAC-seq analysis of NLRP3 promoter region of CRC cell lines is predicted by the Cistrome Data Browser.**
