# Supplementary material for: Inhibition of HDAC2 sensitises antitumour therapy by promoting NLRP3/GSDMD‐mediated pyroptosis in colorectal cancer
Source: Clin Transl Med. 2024 May 28;14(6):e1692. doi: 10.1002/ctm2.1692 (PMC11131357; doi:10.1002/ctm2.1692)
Supplement: Supplementary file 11 — Supporting information [file CTM2-14-e1692-s004.docx]

**
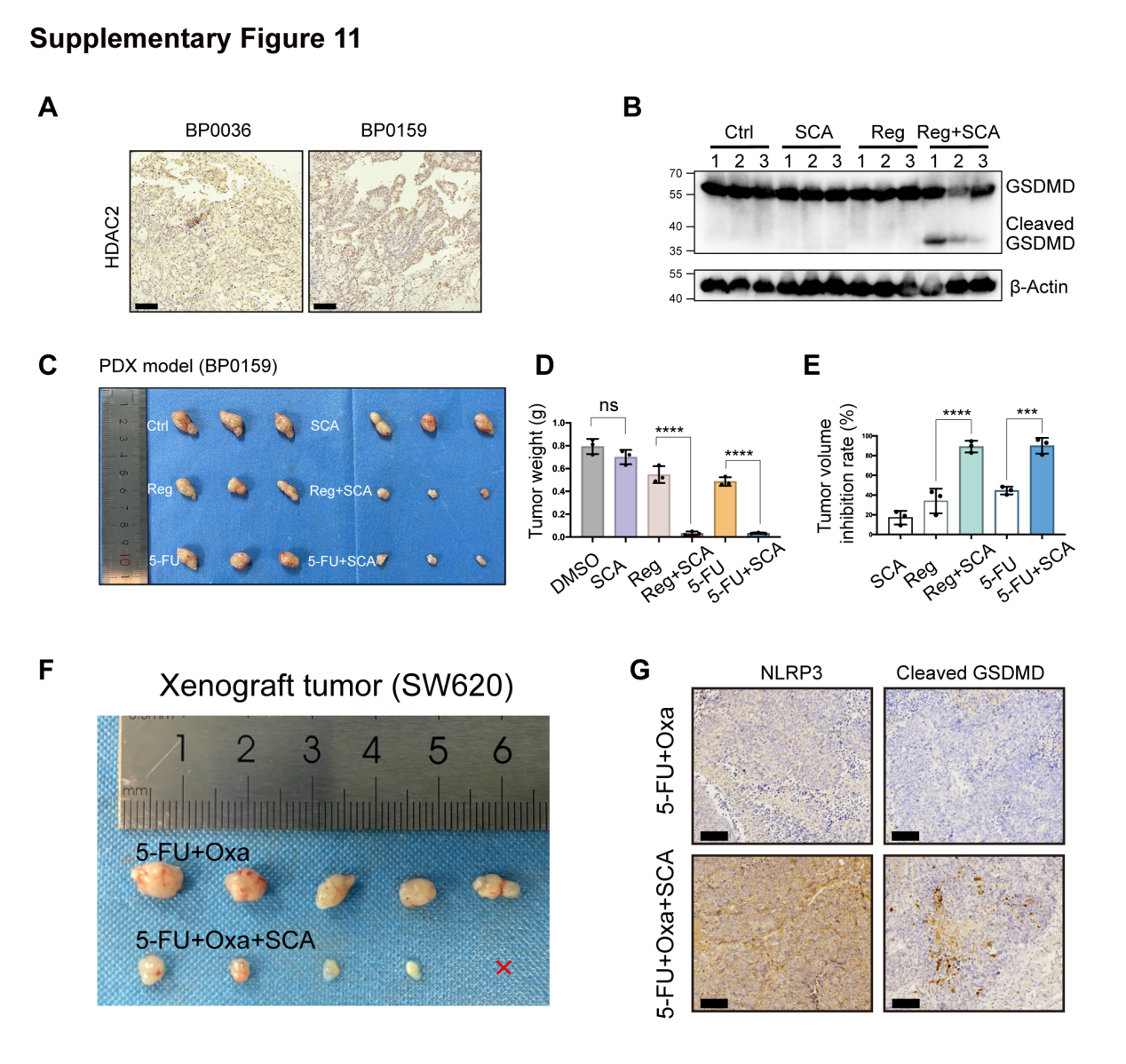
**

**Fig. S11 Inhibition of HDAC2 can sensitize CRC to antitumor therapy. A**Representative IHC staining images showed HDAC2 expression in the BP0036 and BP0159 PDX model. Scale bar: 100 μm. **B** Expression of GSDMD and Cleaved GSDMD in tumor tissues from BP0159 under different treatment conditions was analyzed by western blot. **C, D** In the colorectal cancer PDX model BP0159, which exhibits high expression of HDAC2, tumor size (C) and weight (D) was shown after grouping and 12 days of treatment. **E** Tumor growth inhibition rate of each PDX model under different treatment regimens. **F** SW620 xenografts were established in nude mice and treated as shown (n=5/group). Oxaliplatin is abbreviated as Oxa. **G** The expression of NLRP3 and cleaved GSDMD in xenografts was analyzed by IHC. Scale bar: 100 μm.
