# Supplementary material for: Inhibition of HDAC2 sensitises antitumour therapy by promoting NLRP3/GSDMD‐mediated pyroptosis in colorectal cancer
Source: Clin Transl Med. 2024 May 28;14(6):e1692. doi: 10.1002/ctm2.1692 (PMC11131357; doi:10.1002/ctm2.1692)
Supplement: Supplementary file 12 — Supporting information [file CTM2-14-e1692-s013.docx]

**
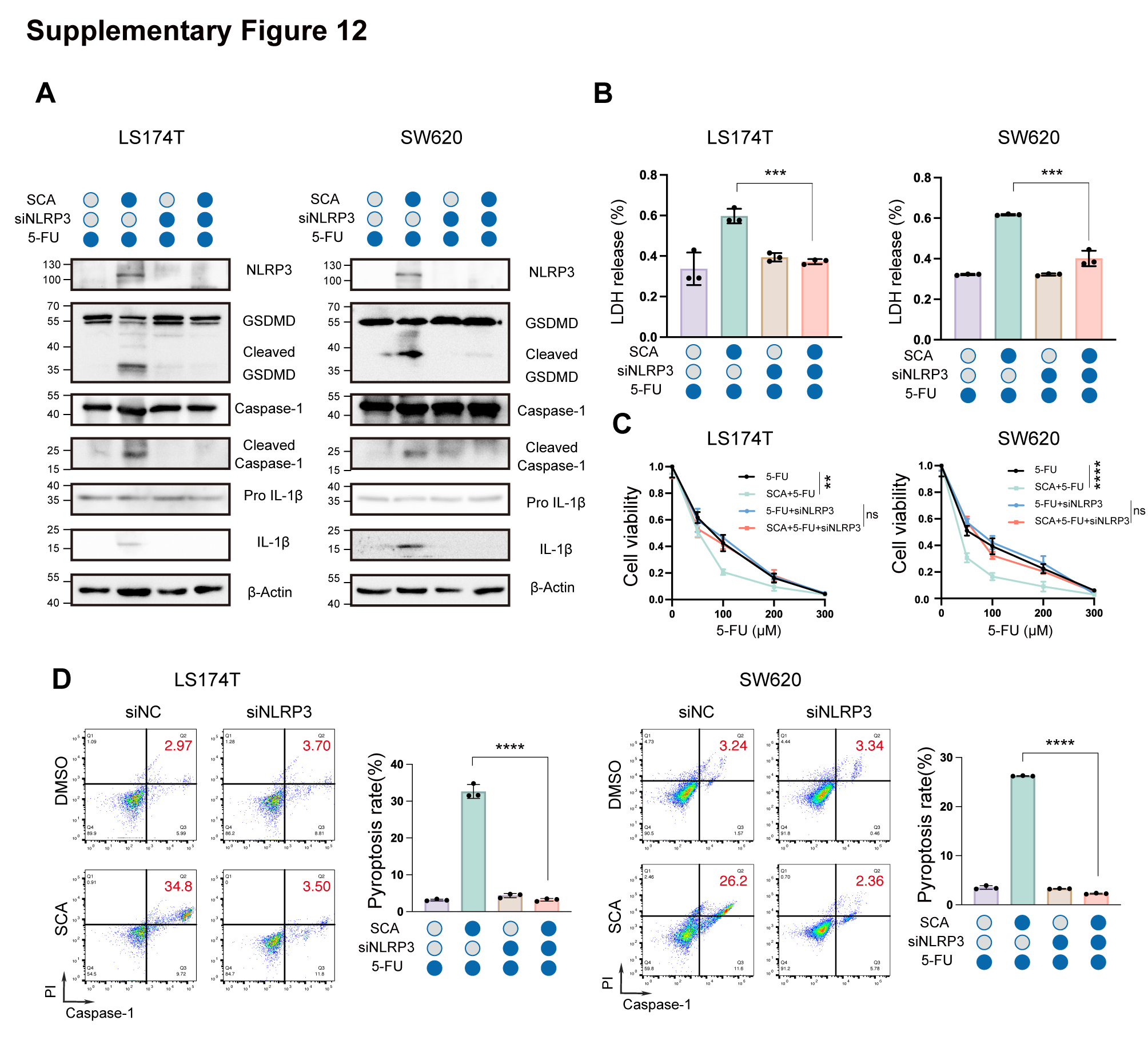
**

**Fig. S12 siRNA-mediated NLRP3 knockdown potently rescued the pyroptosis induced by HDAC2 inhibitor treatment. A** LS174T and SW620 were treated with SCA, and transfected with NLRP3 siRNA for rescue experiments. Pyroptosis pathway proteins were analyzed by western blot. **B** pyroptosis was evaluated by measuring LDH release. **C** A CCK-8 assay was utilized to analyze cell viability across various treatment groups. **D** Flow cytometry analysis for activated Caspase-1/PI.
