# Supplementary material for: Inhibition of HDAC2 sensitises antitumour therapy by promoting NLRP3/GSDMD‐mediated pyroptosis in colorectal cancer
Source: Clin Transl Med. 2024 May 28;14(6):e1692. doi: 10.1002/ctm2.1692 (PMC11131357; doi:10.1002/ctm2.1692)
Supplement: Supplementary file 13 — Supporting information [file CTM2-14-e1692-s001.docx]

**
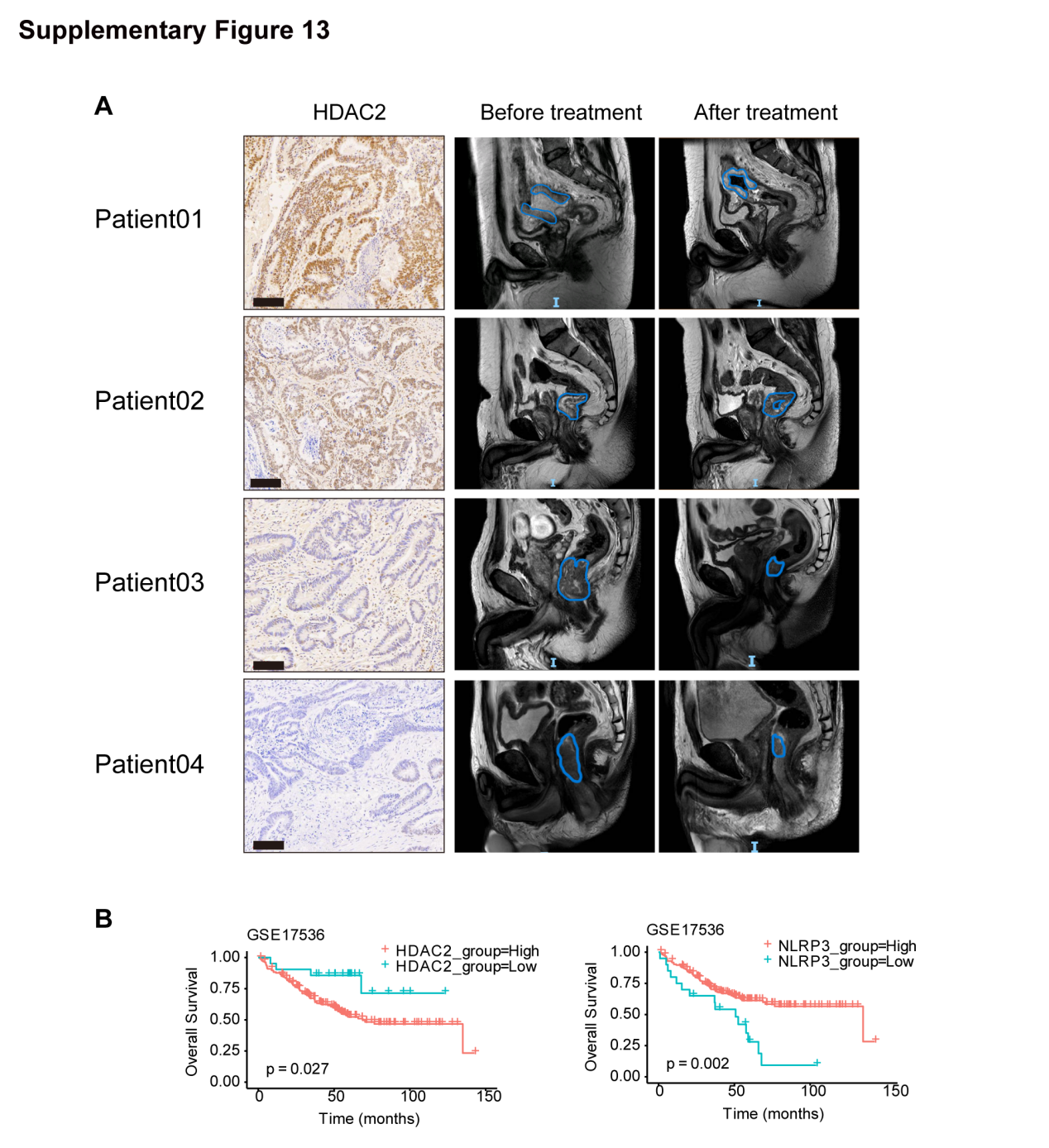
**

**Fig. S13 HDAC2 is a prognostic indicator and holds potential as a CRC therapeutic target. A** The expression of HDAC2 in tumor tissues of neoadjuvant patients with rectal cancer was evaluated by IHC. Scale bar: 100 μm. MRI images of patients with rectal cancer before and after neoadjuvant treatment. **B** Overall survival of colorectal cancer patients classified by HDAC2 and NLRP3 status was analyzed using Kaplan-Meier survival curves in the GSE17536 dataset. statistical significance was assessed by log-rank test.
