# Supplementary material for: Inhibition of HDAC2 sensitises antitumour therapy by promoting NLRP3/GSDMD‐mediated pyroptosis in colorectal cancer
Source: Clin Transl Med. 2024 May 28;14(6):e1692. doi: 10.1002/ctm2.1692 (PMC11131357; doi:10.1002/ctm2.1692)
Supplement: Supplementary file 14 — Supporting information [file CTM2-14-e1692-s010.docx]

| **Antibody** | **Supplier, Clone** | **Application** | **RRID** |
| --- | --- | --- | --- |
| NLRP3 | **Adopigen, Cryo-2** | WB, IHC | unavailable |
| GSDMD | Cell Signaling Technology, E8G3F | WB | # 97558, RRID:AB_2864253 |
| β-actin | Cell Signaling Technology, 13E5 | WB | # 5125, RRID:AB_1903890 |
| Caspase1 | Cell Signaling Technology, D7F10 | WB | # 3866, RRID:AB_2069051 |
| IL-1β | Cell Signaling Technology, D3U3E | WB | # 12703 , RRID:AB_2737350 |
| Cleaved IL-1β | Cell Signaling Technology, D3A3Z | WB | # 83186, RRID:AB_2800010 |
| HDAC2 | Cell Signaling Technology, D6S5P | WB | # 57156, RRID:AB_2756828 |
| NF-κB p65 | Cell Signaling Technology, D14E12 | WB, ChIP | # 8242, RRID:AB_10859369 |
| Phospho-NF-κB p65 (Ser536) | Cell Signaling Technology, 93H1 | WB, IF, IP | # 3033, RRID:AB_331284 |
| BRD4 | Cell Signaling Technology, E2A7X | WB, IP | # 13440, RRID:AB_2687578 |
| GSK-3β | Cell Signaling Technology, D5C5Z | WB | # 12456, RRID:AB_2636978 |
| Phospho-GSK-3β (Ser9) | Cell Signaling Technology, D85E12 | WB | # 5558, RRID:AB_10013750 |
| H3K27ac | Abcam, EP16602 | WB, ChIP, IHC | # ab177178, RRID:AB_2828007 |
| Histone H3 | Abcam, polyclonal | WB | # ab1791, RRID:AB_302613 |
| GSDMD | Abcam, EPR20859 | IHC | # ab219800, RRID:AB_2888940 |
| Cleaved GSDMD | Cell Signaling Technology, E7H9G | IHC | # 36425, RRID:AB_2799099 |
| Cleaved Caspase1 | Invitrogen, polyclonal | IHC | unavailable |
| NF-κB p65 (phospho, S536) | Abcam, polyclonal | IHC | # ab86299, RRID:AB_1925243 |
| H3K27ac | Invitrogen, A6D7 | IF | unavailable |
| HDAC2 | Cell Signaling Technology, 3F3 | IF | # 5113, RRID:AB_10624871 |
| H3K9ac | Abcam, Y28 | ChIP | # ab32129, RRID:AB_732920 |
| IgG | Abcam Cat# ab171870 | ChIP | # ab171870, RRID:AB_2687657 |

**Supplemental Table 1.** Antibodies used in experiments.
