# Supplementary material for: Inhibition of HDAC2 sensitises antitumour therapy by promoting NLRP3/GSDMD‐mediated pyroptosis in colorectal cancer
Source: Clin Transl Med. 2024 May 28;14(6):e1692. doi: 10.1002/ctm2.1692 (PMC11131357; doi:10.1002/ctm2.1692)
Supplement: Supplementary file 15 — Supporting information [file CTM2-14-e1692-s015.docx]

| **Category** | **N (%)** |
| --- | --- |
| Gender  Male  Female | 110  90 |
| Age  ＜60 years  ＞60 years | 83  117 |
| Histological differentiation  Poorly  Moderately  Well | 36  153  11 |
| Depth of invasion  T1  T2  T3  T4 | 3  9  167  21 |
| Lymph node metastasis  N0  N1  N2 | 162  23  15 |
| Metastasis  M0  M1 | 196  4 |
| Pathologic stage  I  II  III  IV | 10  148  39  3 |
| MSI status  MSS  MSI-H | 162  38 |

**Supplemental Table 2.** Clinical characteristics of the 200 CRC patients.
