# Supplementary material for: Inhibition of HDAC2 sensitises antitumour therapy by promoting NLRP3/GSDMD‐mediated pyroptosis in colorectal cancer
Source: Clin Transl Med. 2024 May 28;14(6):e1692. doi: 10.1002/ctm2.1692 (PMC11131357; doi:10.1002/ctm2.1692)
Supplement: Supplementary file 16 — Supporting information [file CTM2-14-e1692-s005.docx]

| **Application** | **Gene** |  | **Sequences** |
| --- | --- | --- | --- |
| Quantitative | H-NLRP3 | Forward  Reverse | CCACAAGATCGTGAGAAAACCC  CGGTCCTATGTGCTCGTCA |
|  | h-GSDMD | Forward  Reverse | GTGTGTCAACCTGTCTATCAAGG  CATGGCATCGTAGAAGTGGAAG |
|  | h-GSDME | Forward  Reverse | CCCAGGATGGACCATTAAGTGT  GGTTCCAGGACCATGAGTAGTT |
| ChIP qPCR | h-NLRP3 promoter-1 | Forward  Reverse | CAGAAGCAAAGAGCCAGAG  TGACCCATCAGCAAGAAAA |
|  | h-NLRP3 promoter-2 | Forward  Reverse | CCGTTATTTGGGTTGATG  ATTGCCTGCTACTTCTTT |

**Supplemental Table 3.** Primers for PCR.
