# Supplementary material for: Inhibition of HDAC2 sensitises antitumour therapy by promoting NLRP3/GSDMD‐mediated pyroptosis in colorectal cancer
Source: Clin Transl Med. 2024 May 28;14(6):e1692. doi: 10.1002/ctm2.1692 (PMC11131357; doi:10.1002/ctm2.1692)
Supplement: Supplementary file 17 — Supporting information [file CTM2-14-e1692-s009.docx]

**Supplementary Methods**

**Cell culture**

HIEC-6, an epithelial cell that was isolated from the small intestine, and human colorectal cancer cell lines (DLD1, HCT116, HT29, RKO, LS174T, HCT15, LoVo, SW620) were acquired from the Cell Bank of the Type Culture Collection, Chinese Academy of Sciences (Shanghai, China). Cells were maintained in RPMI 1640 (NCM460, DLD1), McCoy's 5A (HCT116 and HT29), MEM (RKO, LS174T), DMEM (HCT15), F-12K (LoVo), and L15 (SW620) media. Each medium was supplemented with 10% fetal bovine serum (FBS, Gibco) and 1% penicillin/streptomycin antibiotics (Gibco) while being maintained at 37°C in an atmosphere containing 5% CO2. Short tandem repeat (STR) DNA fingerprinting was used to authenticate all cell lines. Furthermore, rigorous tests ensured the absence of mycoplasma contamination in cell cultures.

**Western blot**

Cultured cells were lysed using ice-cold lysis buffer to extract total protein. Proteins were separated using 10% SDS-PAGE and subsequently transferred to PVDF membranes. Prior to antibody binding, the membranes were blocked in a solution containing 1% Tween-20 in Tris-buffered saline (TBS) and 5% nonfat dry milk. The membranes were then probed with primary antibodies. Following primary incubation, membranes were incubated with secondary anti-mouse/rabbit HRP-conjugated antibodies. Immunoreactive bands were visualized using a chemiluminescence agent (Thermo Fisher Scientific).

**Immunohistochemistry (IHC)**

Paraffin-embedded tissue sections were deparaffinized, and antigen retrieval was performed in a water bath with 0.01 M sodium citrate buffer. Endogenous peroxidase activity was inactivated in 5% hydrogen peroxide solution for 30 min at room temperature. Sections were then blocked in 3% bovine serum albumin (BSA) for 30 min at room temperature, followed by overnight incubation with primary antibodies at 4°C. After washing with PBS, the sections were incubated for 30 min with anti-rabbit and anti-mouse antibodies. The chromogenic reaction was performed using DAB (Thermo Shandon), and sections were counterstained with hematoxylin. All the slides were digitized with a ×20 objective using a Motic EasyScan slide scanner (Motic Europe, Barcelona, Spain). All whole-slide images were viewed using the Motic DSAssistent software.

**Dual-luciferase reporter assay**

After seeding colorectal cancer cells in 24-well plates, NF-κB, STAT1, STAT3, NFATC1, and AP-1 activity reporter plasmids (Beyotime Biotechnology, Wuhan, China) were co-transfected with a Renilla luciferase expression plasmid (Promega). Following transfection, cells were incubated with or without 10µM regorafenib for 24h. Dual-luciferase reporter assays were performed using the Dual-Luciferase Reporter Assay System (Promega), following the manufacturer's instructions as well as previous methods. Firefly luciferase activity was normalized to the corresponding Renilla luciferase activity to ensure the transfection efficiency. Relative promoter activity was determined by the luminescence ratio of firefly to Renilla.

**Cell viability assay**

CRC cells were seeded in 96-well plates at a density of 4000 cells per well. After 24h, cells were treated with the indicated reagents. Viability was then assessed 48h post-treatment using Cell Counting Kit-8 (CCK-8) reagent (KeyGEN) following the manufacturer's instructions. The absorbance of each well was measured at a wavelength of 450 nm.

**Total RNA extraction and real-time quantitative PCR**

Total RNA was extracted from cultured cells using TRIzol reagent, as previously reported. Briefly, 2mg of total RNA was reverse-transcribed using a cDNA synthesis kit (Takara). Real-time quantitative PCR (qPCR) was then carried out in a 10 μL reaction containing cDNA (30 ng), SYBR-Green mix, and primers. The amplified transcript level of each gene was normalized to that of β-actin using the 2-ΔΔCt method. A detailed list of the primer sequences used in this study is shown in Table S3.

**Mouse colon cancer lung metastasis model**

To establish the lung metastasis model, BALB/c nude female mice aged 6-8 weeks were intravenously injected with 4 x 10^6 SW620 HDAC2 knockout cells suspended in PBS via the tail vein. Three days after injection, mice were randomly assigned to different treatment groups: 5-FU (25 mg/kg, twice a week), regorafenib (30 mg/kg, daily), 5-FU (25 mg/kg kg, twice a week) combined with Dehydroxymethylepoxyquinomicin (DHMEQ) (12 mg/kg, three times a week), regorafenib (30 mg/kg, daily) combined with DHMEQ (12 mg /kg, three times a week). Treatment lasted for 2 weeks, and mouse body weight was monitored daily. At the end of the experiment, the mice were euthanized, and the lungs were excised to quantify the number of metastatic tumor nodules present. Lung sections were prepared, stained with H&E, and the number of lung metastatic nodules was quantified. All experimental procedures were approved by the Ethics Committee of Harbin Medical University.

**Orthotopic mouse model of colorectal cancer**

Six-week-old female BALB/c nude mice underwent a minimally invasive midline laparotomy to expose the cecum. Mice were anesthetized for this procedure to ensure minimal discomfort. The cecum was gently exteriorized, and SW620 wild-type or HDAC2 KO colorectal cancer cells (1×10^6 cells) suspended in serum-free DMEM were injected into the cecal wall using a microsyringe. The cecum was then carefully returned to the abdominal cavity. Following tumor establishment, mice were treated with regorafenib or 5-FU for 2 weeks, with the same dosage and usage as described above. All interventions were maintained for one month, after which mice were euthanized humanely.

**Hematoxylin and Eosin (H&E) Staining Assay**

The formalin-fixed, paraffin-embedded tissue sections were deparaffinized through a series of xylene baths and rehydrated in graded ethanol solutions. Following rehydration, the sections were stained with Mayer's hematoxylin solution for 5-8 minutes to label the nuclei with a deep blue-purple color. Excess hematoxylin was removed by rinsing in tap water until the sections exhibited a desired stain intensity. The sections were then counterstained with eosin Y solution (aqueous or alcoholic) for 1-3 minutes to stain the cytoplasmic components shades of red, pink, and orange. Afterward, the sections were dehydrated through graded ethanol solutions, cleared in xylene, and permanently mounted with a xylene-based mounting medium for microscopic examination and imaging.
